# Salivary Gland Dysfunction in Patients with Chronic Heart Failure Is Aggravated by Nitrosative Stress, as Well as Oxidation and Glycation of Proteins

**DOI:** 10.3390/biom11010119

**Published:** 2021-01-18

**Authors:** Anna Klimiuk, Anna Zalewska, Małgorzata Knapp, Robert Sawicki, Jerzy Robert Ładny, Mateusz Maciejczyk

**Affiliations:** 1 Experimental Dentistry Laboratory, Medical University of Bialystok, 24a M. Sklodowskiej-Curie Street, 15-274 Bialystok, Poland; annak04@poczta.onet.pl (A.K.); azalewska426@gmail.com (A.Z.); 2 Department of Cardiology, Medical University of Bialystok, 24a M. Sklodowskiej-Curie Street, 15-274 Bialystok, Poland; malgo33@interia.pl (M.K.); r-sawicki@o2.pl (R.S.); 3 1st Department of General Surgery and Endocrinology, Medical University of Bialystok, 24a M. Sklodowskiej-Curie Street, 15-274 Bialystok, Poland; ladnyjr@wp.pl; 4 Department of Hygiene, Epidemiology and Ergonomics, Medical University of Bialystok, 2c Mickiewicza Street, 15-233 Bialystok, Poland

**Keywords:** chronic heart failure, salivary gland dysfunction, protein oxidation, protein glycation

## Abstract

Chronic heart failure (HF) is an important clinical, social, and economic problem. A key role in HF progression is played by oxidative stress. Free oxygen radicals, formed under the conditions of hypoxia and reperfusion, participate in myocardial stunning and other forms of post-reperfusion damage. HF patients also suffer from disorders connected with saliva secretion. However, still little is known about the mechanisms that impair the secretory function of salivary glands in these patients. In the presented study, we were the first to compare the antioxidant barrier, protein glycoxidation, and nitrosative/nitrative stress in non-stimulated (non-stimulated whole saliva (NWS)) and stimulated (SWS) saliva of HF patients. The study included 50 HF patients with normal saliva (NS) secretion (*n* = 27) and hyposalivation (HS) (*n* = 23), as well as an age- and gender-matched control group (*n* = 50). We demonstrated that, in NWS of HF patients with HS, the concentration of low-molecular-weight non-enzymatic antioxidants decreased (↓total polyphenols, ↓ascorbic acid, ↓reduced glutathione, ↓albumin) compared to HF patients with normal saliva (NS) secretion, as well as the control group (except albumin). We also observed increased content of protein glycoxidation products (↑dityrosine, ↑kynurenine, ↑glycophore) in NWS and SWS of HF patients with HS compared to healthy controls. Interestingly, the content of dityrosine, N-formylkynurenine, and glycophore in NWS was also significantly higher in HF patients with HS compared to those with NS secretion. The concentration of NO was considerably lower, while the levels of peroxynitrite and nitrotyrosine were significantly higher in NWS and SWS of HF subjects with HS compared to the controls. Salivary gland dysfunction occurs in patients with chronic HF with the submandibular salivary glands being the least efficient. Oxidative/nitrosative stress may be one of the mechanisms responsible for the impairment of salivary gland secretory function in HF patients.

## 1. Introduction

Chronic heart failure (HF) is a pathological condition in which the heart cannot deliver sufficient amount of blood to tissues and organs according to their current metabolic needs [1,2]. HF affects 1–2% of the population in developed countries and is currently one of the main causes of death worldwide. Thus, HF is not only a significant medical problem but also a social one [3]. The most common HF risk factors include hypercholesterolemia, hypertension, smoking, diabetes, unbalanced diet, stress, and sedentary lifestyle [4,5,6]. On a molecular level, HF is defined as a defect of contractile proteins and myocyte organelles, as well as humoral disorders and changes in the cardiovascular and nervous systems that occur during heart damage in the course of various systemic diseases. Indeed, the occurrence of HF separately is rare in clinical practice [7]. Patients with HF often suffer from two or more conditions simultaneously, particularly as the incidence of concomitant diseases increases with age [8]. As a consequence, polypharmacotherapy is required, which often leads to numerous side effects, including those that also affect the oral cavity. In patients taking cardiological drugs (e.g., beta blockers, angiotensin-converting enzyme (ACE) inhibitors, and diuretics), we can observe reduced saliva production (hyposalivation (HS)), abnormal protein secretion into the saliva, and a subjective sensation of dry mouth (xerostomia) [9,10].

However, not only pharmacotherapy but also a number of systemic diseases can affect salivary gland activity. Reduced saliva secretion has been observed in patients with hypertension [11,12], chronic kidney disease [13,14], obesity [15,16], diabetes [17,18], psoriasis [19,20], and dementia [21,22]. It is believed that oxidative/nitrosative stress is a key factor leading to progressive salivary gland failure. In a state of decreased capacity of antioxidant systems, the intensity of oxidation/nitration of cellular biomolecules is boosted. As a result, these biomolecules are aggregated and accumulated in salivary glands, thus hindering saliva secretion [14,23]. Disorders in the quantitative and qualitative composition of saliva entail numerous pathological consequences [24,25]. This fact is not surprising as saliva has a considerable impact on human health: it participates in food digestion, ensures proper hydration of the oral mucosa, removes harmful metabolic products, bacteria and viruses, and is necessary for the remineralization of hard dental tissues. Additionally, saliva maintains the redox balance in the oral cavity and participates in the body’s immune response [26,27,28]. However, still little is known about the secretory dysfunction of salivary glands in HF patients. Considering the key role of oxidative/nitrosative stress in the pathogenesis of HF (myocardial and blood vessel damage) [29,30], it can be assumed that this process is also involved in salivary gland hypofunction. Numerous studies have demonstrated that peroxynitrite, which is generated in blood vessels, is a potent oxidant responsible for the nitration of aromatic amino acid residues (such as tryptophan and tyrosine), as well as the decrease in antioxidant barrier capacity [31,32].

In our previous study, we showed disturbances in enzymatic and non-enzymatic antioxidant systems, as well as enhanced oxidative lipid damage in saliva and plasma/erythrocytes of HF patients [33]. Disturbances in redox homeostasis generally worsen with disease progression, and some salivary biomarkers may have a diagnostic potential [33]. However, in HF patients, the contribution of oxidative/nitrosative stress to salivary gland damage is still unknown. Since HS significantly reduces the quality of life of patients with chronic HF, it is essential to understand the mechanisms that lead to salivary gland dysfunction in the course of HF. Therefore, the aim of our research was to assess the relationship between the degree of salivary gland damage and redox homeostasis in HF patients with normal salivary secretion, as well as HS. In the non-stimulated saliva (non-stimulated whole saliva (NWS)), stimulated saliva (SWS), plasma, and erythrocytes of HF patients and healthy controls, we assessed, the concentration of low-molecular-weight antioxidants, redox status, content of glycoxidation products, and nitrosative stress biomarkers. To evaluate the secretory function of salivary glands, we measured the salivary flow rate, total protein content, and salivary amylase activity.

## 2. Materials and Methods

### 2.1. Ethical Issues

The study was approved by the Bioethics Committee of the Medical University of Bialystok, Poland (permission number R-I-002/75/2016). All persons participating in the study gave their written consent to participate in the experiment after obtaining a thorough explanation of the purpose of the study and possible risks connected with it.

### 2.2. Patients

Patients with chronic HF, hemodynamically stable, qualified for the implantation of an automatic implantable cardioverter-defibrillator or the cardiac resynchronization therapy system were included in the study (Table 1). The qualification criterion for the procedure was left ventricular ejection fraction (LVEF) < 35%. The study group consisted of 50 patients treated in the Department of Cardiology with the Intensive Cardiac Care Unit of the Medical University of Bialystok Clinical Hospital. The patients were divided into two subgroups based on their flow of non-stimulated saliva (NWS): a group with normal saliva (NS) secretion (HF NS) and one with reduced saliva secretion (hyposalivation (HS); HF HS). Hyposalivation was defined as an NWS flow below 0.2 mL/min [11,14,20].

The control group, selected by gender and age to match the study group, consisted of 50 generally healthy participants who reported for follow-up visits to the Outpatient Clinic of Conservative Dentistry of the Medical University of Bialystok Specialized Dental Clinic. All subjects from the control group had an NWS flow above 0.2 mL/min.

Patients with body mass index (BMI) between 18.5 and 24.5 were qualified for the study and the control groups. The exclusion criterion in both groups was the presence of chronic systemic and autoimmune diseases (type 1 diabetes mellitus, Sjögren’s syndrome, rheumatoid arthritis, psoriasis), lung, thyroid, liver, kidney, digestive tract, or infectious diseases (HCV, HBV, HIV infection), as well as immunological disorders. Moreover, the study did not involve subjects with periodontal disease, smokers, alcoholics, and patients taking antibiotics, non-steroidal anti-inflammatory drugs, glucocorticosteroids, vitamins, and dietary supplements within 3 months prior to the experiment.

### 2.3. Research Material

The research material, which consisted of venous blood and total non-stimulated (NWS) and stimulated (SWS) saliva collected via the spitting method, was obtained from patients before the implantation of an automatic cardioverter-defibrillator or the resynchronization system.

### 2.4. Blood Collection

Venous blood (10 mL) was collected from the subjects after an overnight rest, on an empty stomach, using the S-Monovette^®^ K3 EDTA blood collection system (Sarstedt, Nümbrecht, Germany). The blood samples were then centrifuged (1500× *g*, 10 min, +4 °C; MPW 351, MPW Med. Instruments, Warsaw, Poland). Only the samples without any signs of hemolysis were qualified for further testing. The upper layer—plasma—was taken, and erythrocytes were rinsed three times with 0.9% NaCl cold solution and hemolyzed by adding 9 volumes of cold 50 mM phosphate buffer [34]. To protect the samples against oxidation, butylated hydroxytoluene (BHT) antioxidant was added [35]. The samples were stored at −80 °C for no longer than 6 months.

### 2.5. Saliva Collection

In order to minimize the effect of the daily rhythm on saliva secretion, the samples were collected in the morning, between 8 a.m. and 10 a.m., with any additional stimuli eliminated. Two hours prior to saliva collection, the subjects from the study/control group refrained from consuming any food or beverages (excluding clean water), as well as from oral hygiene procedures. Moreover, they had not taken any medications at least 8 h before saliva collection [36,37]. After rinsing their mouth three times with distilled water at room temperature, the participants spit saliva accumulated at the bottom of the oral cavity into a sterile Falcon tube (cooled in a container with ice). The saliva collected during the first minute was discarded. NWS was collected for 10 min. After a 5-min break, SWS was collected for 5 min up to a maximum volume of 5 mL (upon stimulation by applying 10 µL 2% citric acid on the tip of the tongue every 30 s). The collected saliva was immediately centrifuged (3000× *g*, 20 min, +4 °C) [38]. Butylated hydroxytoluene (5 μL 0.5 M BHT in acetonitrile per 0.5 mL of salivary supernatant) was added to the obtained supernatants to protect them against oxidation processes. The samples were stored at −80 °C for no longer than six months [35].

### 2.6. Dental Examination

Immediately after non-stimulated and stimulated saliva collection, the subjects had the dental examination performed by the same dentist (A.K.) each, according to the criteria of the World Health Organization: in artificial lighting, using a mirror, an explorer, and a periodontal probe [39]. DMFT (decay, missing, filled teeth), PBI (Papilla Bleeding Index), GI (Gingival Index), and the occurrence of carious lesions of root cement (CR) were determined. The DMFT index is the sum of teeth with caries (D), teeth extracted because of caries (M), and teeth filled because of caries (F). The PBI showed the intensity of bleeding from the gingival papilla after probing [40]. GI criteria include qualitative changes in the gingiva [41]. Inter-rater agreements were assessed in 30 patients. The reliability for DMFT was r = 0.96, for PBI: r = 0.96, and for GI: r = 0.99.

### 2.7. Total Protein

The concentration of total protein was determined colorimetrically with a commercial kit Thermo Scientific PIERCE BCA Protein Assay (Rockford, IL, USA) according to the bicinchoninic method in which bicinchoninic acid (BCA) reacts with copper ions (2+), forming a stable complex that shows a maximum absorption at 562 nm wavelength. The concentration of total protein was expressed in μg/mL.

### 2.8. Salivary Amylase

The activity of salivary amylase (EC 3.2.1.1) was determined colorimetrically at 540 nm wavelength, using 3,5-dinitrosalicylic acid (DNS). We also measured absorbance changes accompanying the increased concentration of reducing sugars that were released during hydrolysis of starch, catalyzed by salivary amylase [35,42]. The activity of salivary amylase was determined in duplicate samples and expressed in μg/mg total protein.

### 2.9. Biochemical Assays

The levels of non-enzymatic antioxidants, redox status, protein glycoxidation products, and nitrosative stress biomarkers were determined in saliva samples, as well as plasma/erythrocytes. Reagents for all the said assays (unless stated otherwise) were purchased from Sigma-Aldrich, Nümbrecht, Germany or Sigma-Aldrich, Saint Louis, MO, USA. The absorbance/fluorescence of the samples was measured with the Infinite M200 PRO microplate reader (Tecan Group Ltd., Männedorf, Switzerland). All results were standardized to 1 mg of total protein.

### 2.10. Salivary Antioxidants

The total polyphenol content (TPC) was determined by the colorimetric method using the Folin-Ciocalteu (FC) reagent, which is a mixture of phosphotungstic acid and phosphomolybdic acid. By reacting with phenols, FC releases a blue product with a maximum absorption spectrum at 760 nm. The content of TPC was calculated from the standard curve for gallic acid (GAE) and expressed as μg/mg total protein. The determinations were performed in duplicate samples.

The concentration of ascorbic acid (AA) was determined colorimetrically using FC. The absorption maximum of the color developed by the interaction of AA with FC was 760 nm [43]. The assays were performed in duplicate samples and expressed in μg/mg total protein.

Uric acid concentration (UA) was determined colorimetrically using a ready-made BioAssay System reagent kit (QuantiChrom TM Uric Acid Assay Kit DIUA-250, BioAssay System, Hayward, CA, USA). The method is based on the reaction of 2,4,6- tripyridyl-s-triazine with iron ions (3+) in the presence of UA contained in the examined sample. Absorbance changes of the resulting complex were measured at 590 nm wavelength. The determinations were performed in duplicate samples and expressed in μg/mg total protein.

The concentration of reduced glutathione (GSH) was assayed by the colorimetric method based on the reduction of 5,5′-dithiobis-(2-nitrobenzoic acid) (DTNB) to 2-nitro-5-mercaptobenzoic acid under the influence of GSH contained in the sample. The absorbance changes were measured at 412 nm wavelength [44]. The determinations were performed in duplicate samples and expressed in μg/mg total protein.

Albumin concentration was measured colorimetrically using bromocresol green. The addition of albumin to the bromocresol green solution in succinate buffer resulted in increased absorbance at 628 nm wavelength. The assays were performed in duplicate samples and expressed in mg/mg total protein.

### 2.11. Salivary Redox Status

The total antioxidant activity of every sample was evaluated using the DPPH (1,1-diphenyl-2-picrylhydrazyl radical) reduction method [45]. In the presence of antioxidants, DPPH^∙^ is discolored, which is the basis for the colorimetric measurement at 515 nm wavelength. The determination of DPPH was performed in triplicate samples and was expressed in nmol/mg total protein.

The ability to reduce iron ions (ferric-reducing antioxidant power (FRAP)) was determined colorimetrically based on the reduction of Fe^3+^-TPTZ complex (2,4,6-tripyridyl-s-triazine complex of iron (III)) to Fe^2+^-TPTZ under the influence of antioxidants contained in the assayed sample. The resulting complex reached its maximum absorption at 593 nm wavelength. FRAP concentration was calculated from the standard curve for iron (2+) sulphate and expressed as µmol/mg total protein [46]. FRAP determination was performed in triplicate samples.

### 2.12. Salivary Glycoxidation Products

In order to evaluate the content of glycoxidatively modified proteins (dityrosine, kynurenine, N-formylkynurenine, and tryptophan), saliva samples were diluted in 0.1 M sulfuric acid at a volume ratio of 1:10 [22]. After thorough mixing, fluorescence of the samples was measured at wavelengths of: 330/415 (dityrosine), 365/480 (kynurenine), 325/434 (N-formylkynurenine), and 95/340 (tryptophan). The content of glycoxidatively modified amino acids was expressed in arbitrary fluorescence units (AFU)/mg of total protein [34,47]. All determinations were performed in duplicate samples.

The formation of glucose-derived fluorescence, termed glycophore, was determined fluorimetrically. The principle of this method is to measure the fluorescence of furoyl-furanyl-imidazole (FFI), carboxymethyl-lysine (CML), pyraline, and pentosidine, typical of advanced glycation end products (AGE) of proteins. Immediately prior to the determination, the samples were diluted in PBS buffer (0.02 M, pH 7.0) at a volume ratio of 1:5 and mixed thoroughly. Fluorescence of the samples was measured at 350 nm excitation wavelength and 440 nm emission wavelength [48]. AGE content was determined in duplicate samples and expressed in AFU/mg total protein.

### 2.13. Salivary Nitrosative Stress

The activity of myeloperoxidase (MPO) was measured colorimetrically at 450 nm wavelength using sulfanilamide, ortho-dianisidine dihydrochloride, hexadecyltrimethylammonium, and hydrogen peroxide [49]. The activity of MPO was determined in duplicate samples and expressed in mU/mg total protein.

Nitric oxide (NO) concentration was assayed by the colorimetric method based on the reaction of nitrates (3+) with sulfanilamide and N-(1-naphthyl)-ethylenediamine dihydrochloride, resulting in the formation of a colored product with a maximum absorption at 490 nm wavelength [50,51]. NO concentration was determined in duplicate samples and expressed in µmol/mg total protein.

Peroxynitrite concentration was determined fluorimetrically by measuring the degree of nitrosylation of phenol. S-nitrophenol, formed as a result of the reaction of peroxynitrite and phenol, exhibited its maximum absorption at 490 nm excitation wavelength and 530 nm emission wavelength. Molar absorption coefficient ε = 1670 M M^−1^ cm^−1^ [52] was used to calculate peroxynitrite concentration, which was assayed in duplicate samples and expressed in µmol/mg total protein.

The concentration of S-nitrosothiols was measured colorimetrically based on the Griess reagent reaction with S-nitrosothiols contained in the tested sample, followed by the reaction with Hg^2+^ mercury ions. The maximum absorption of the resulting complex occurred at 490 nm wavelength. Molar absorption coefficient ε = 11,500 M^−1^ cm^−1^ was used to calculate the concentration of S-nitrosothiols [50,53]. The concentration of S-nitrosothiols was determined in duplicate samples and expressed in µmol/mg total protein.

Nitrotyrosine concentration was determined by ELISA using the Nitrotyrosine ELISA kit from Immunodiagnostik AG (Bensheim, Germany) according to the manufacturer’s instructions. Determinations were performed in duplicate samples and expressed in µmol/mg total protein.

### 2.14. Statistical Analysis

The statistical package GraphPad Prism 8 for Mac (GraphPad Software, La Jolla, CA, USA) was used for data analysis. The distribution of results was checked using the Shapiro-Wilk test and the Kolmogorov–Smirnov test. Due to the lack of normality of the distribution, we used a non-parametric analysis of variance called the Kruskal–Wallis test. The Dunn test was used for multiple comparisons and multiplicity-adjusted p value was calculated. The Mann–Whitney U test was performed to analyze differences between the two groups. The Pearson’s correlation coefficient was used to assess the correlation between the dependent variables. The assessment of the diagnostic utility of redox biomarkers was based on ROC (Receiver Operating Characteristics) curves. The maximum area under curve (AUC), with values from 0 to 1, is a parameter that determines the discriminatory power of the test. The results for *p* < 0.05 were considered statistically significant.

The number of patients was set a priori based on the pilot study. For this purpose, an online sample size calculator (ClinCalc) was used. The minimum number of patients was 37 (level of significance = 0.05; power of study = 0.9).

## 3. Results

### 3.1. Dental Examination and Salivary Gland Function

The secretory activity of salivary glands was analyzed by measuring the salivary flow rate and evaluating the total protein and amylase activity in saliva. The results are summarized in Table 2. We observed significantly lower flow of NWS and SWS in HF patients with normal salivation (NS), as well as HF patients with HS compared to the control, and considerably lower NWS salivary flow in HF patients with HS compared to HF subjects with NS.

Total protein content was significantly lower in HF patients with HS compared to both HF patients with NS and the control group.

The activity of salivary amylase was significantly lower in NWS, as well as SWS, in both study groups of patients compared to healthy controls. Moreover, in NWS of HF patients with HS, the activity of salivary amylase (SA) was considerably lower compared to HF patients with NS.

No significant differences in DMFT, PBI, GI, and CR were found in patients from both the study and control groups.

### 3.2. Salivary Antioxidants

In NWS, the total polyphenol content (↓64.18%, *p* < 0.0001; ↓32.84%, *p* < 0.0001, respectively) and the concentration of AA (↓77.78%, *p* = 0.0083; ↓50%, *p* < 0.0001, respectively) and GSH (↓50%, *p* < 0.0001; ↓25.36%, *p* < 0.0001, respectively) were significantly lower in HF patients with NS and HS compared to the control group, while albumin content (↓45.16%, *p* < 0.0001) was considerably lower only in HF patients with HS. UA concentration in HF subjects with HS was markedly higher compared to the control (↑69.47%, *p* = 0.0383) and HF patients with normal salivary secretion (↑76.84%, *p* < 0.0001). Within the study group, TPC (↑51.16, *p* = 0.0196), as well as the concentration of AA (↑64.29%, *p* = 0.0081), GSH (↑50.71%, *p* = 0.0249), and albumins (↑65%, *p* = 0.0032), were significantly higher in HF patients with NS compared to HS ones with HS.

**Figure 1:**
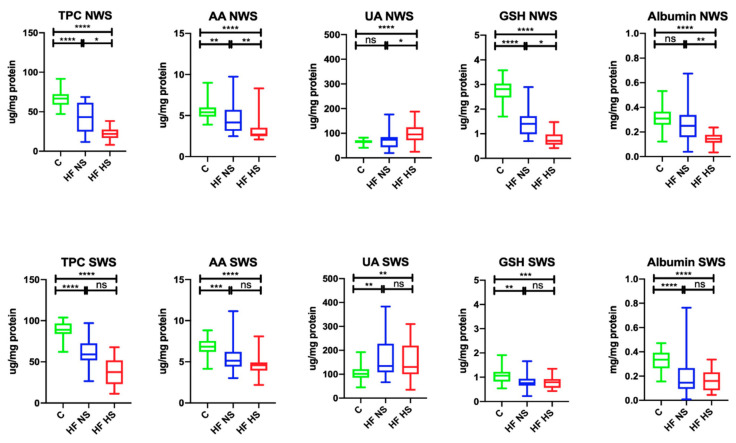
Salivary antioxidants in non-stimulated and stimulated saliva of HF patients and the control group. Abbreviations: AA—ascorbic acid; GSH—reduced glutathione; HF NS—heart failure with normal salivation; HF HS—heart failure with hyposalivation; NWS—non-stimulated whole saliva; Px—salivary peroxidase; SOD—superoxide dismutase-1; SWS—stimulated whole saliva; TPC—total polyphenol content; UA—uric acid; * *p* < 0.05, ** *p* < 0.01, *** *p* < 0.001, and **** *p* < 0.0001.

In SWS, TPC (↓66.29%, *p* < 0.0001; ↓42.7%, *p* < 0.0001, respectively), as well as the concentration of AA (↓75%, *p* < 0.0001; ↓67.64, *p* < 0.0001, respectively), GSH (↓69.09, *p* = 0.0016; ↓71.82%, *p* = 0.0004), and albumins (↓44.12%, *p* < 0.0001; ↓47.06%, *p* < 0.0001, respectively) were considerably lower in HF patients with HS and HS compared to the control group, while UA concentration (↑76.12%, *p* = 0.0015; ↑78.46%, *p* = 0.003, respectively) was significantly higher (Figure 1).

### 3.3. Salivary Redox Status

In NWS, DPPH (↓77.03%, *p* = 0.0044; ↓37.32%, *p* < 0.0001, respectively) and FRAP (↓69.7%, *p* = 0.0008; ↓62.12%, *p* < 0.0001, respectively) were significantly lower in the group of HF subjects with NS and those with HS compared to healthy controls. Within the study group, DPPH (↑48.44%, *p* = 0.0183) and FRAP (↑89.13%, *p* = 0.0281) were considerably higher in HF patients with NS compared to those with HS.

**Figure 2:**
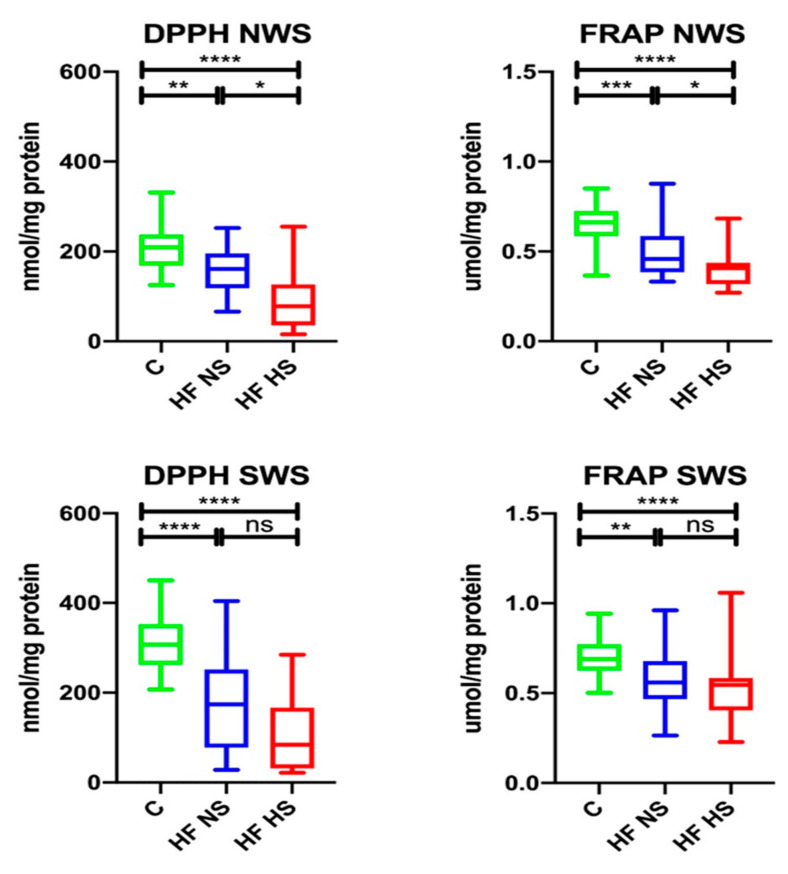
Salivary redox status in non-stimulated and stimulated saliva of HF patients and the control group. Abbreviations: DPPH—2,2-diphenyl-1-picrylhydrazyl radical; FRAP—ferric-reducing antioxidant power; HF NS—heart failure with normal salivation; HF HS—heart failure with hyposalivation; NWS—non-stimulated whole saliva; SWS—stimulated whole saliva; * *p* < 0.05, ** *p* < 0.01, *** *p* < 0.001, and **** *p* < 0.0001.

W SWS, DPPH (↓56.68%, *p* < 0.0001; ↓27.36%, *p* < 0.0001, respectively), and FRAP (↓81.16%, *p* = 0.0021; ↓79.71%, *p* < 0.0001, respectively) were statistically lower in HF patients with NS, as well as HS, compared to the control group (Figure 2).

### 3.4. Salivary Glycoxidation Products

In NWS, the content of dityrosine (↑73.33%, *p* = 0.0004; ↑68.75%, *p* < 0.0001, respectively), kynurenine (in both cases ↑78.95%, *p* < 0.0001), N-formylkynurenine (↑70.71%, *p* < 0.0001; ↑49.5%, *p* < 0.0001, respectively), and glycophore (↑83.33%, *p* < 0.0001; ↑62.5%, *p* < 0.0001, respectively) was significantly higher in the group of HF patients with NS and HS compared to the controls, and the content of tryptophan (↓81.82%, *p* = 0.0475) was considerably lower in the HF HS group in comparison with the control group. Within the study group, the levels of dityrosine (↓93.75%, *p* = 0.0472), N-formylkynurenine (↓70%, *p* = 0.0487) and glycophore (↓75%, *p* = 0.0174) were markedly higher in HF patients with NS compared to HF subjects with HS.

**Figure 3:**
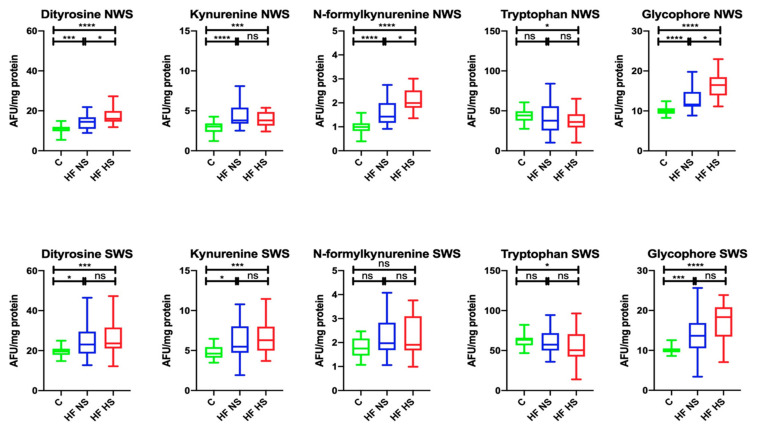
Salivary glycoxidation product status in non-stimulated and stimulated saliva of HF patients and the control group. Abbreviations: HF NS—heart failure with normal salivation; HF HS—heart failure with hyposalivation; NWS—non-stimulated whole saliva; SWS—stimulated whole saliva; * *p* < 0.05, *** *p* < 0.001, and **** *p* < 0.0001.

In SWS, the content of dityrosine (↑86.96%, *p* = 0.0108; ↑95.83%, *p* = 0.0005, respectively), kynurenine (↑83.64%, *p* = 0.0265; ↑73.02%, *p* = 0.0005, respectively) and glycophore (↑71.43%, *p* = 0.0005; ↑55.56%, *p* < 0.0001, respectively) was significantly higher in HF patients with NS and HS compared to the control group, and tryptophan content (↓81.82%, *p* = 0.0215) was considerably lower in the HF HS group than in the controls (Figure 3).

### 3.5. Salivary Nitrosative Stress

In NWS, MPO activity (↑43.48%, *p* < 0.0001; ↑29.41%, *p* < 0.0001, respectively) and the concentration of peroxynitrite (↑60.29%, *p* < 0.0001; ↑41%, *p* < 0.0001, respectively) and nitrotyrosine (↑68.52%, *p* = 0.0011; ↑53.78%, *p* < 0.0001, respectively) were significantly higher in the group of HF patients with NS and HS compared to the controls, while the content of S-nitrosothiols (↑75.61%, *p* = 0.1054) was considerably higher only in HF patients with HS compared to the control group. NO concentration (↓74.64%, *p* = 0.0122; ↓48.21%, *p* < 0.0001, respectively) was markedly lower in the study group (HF patients with NS, as well as HS) than in healthy controls. Within the study group, MPO activity (↓67.65%, *p* = 0.0497) and the concentration of peroxynitrite (↓68%, *p* = 0.049) were significantly lower in HF participants with NS compared to HF patients with HS, while NO concentration (↑64.59%, *p* = 0.0344) was considerably higher.

**Figure 4:**
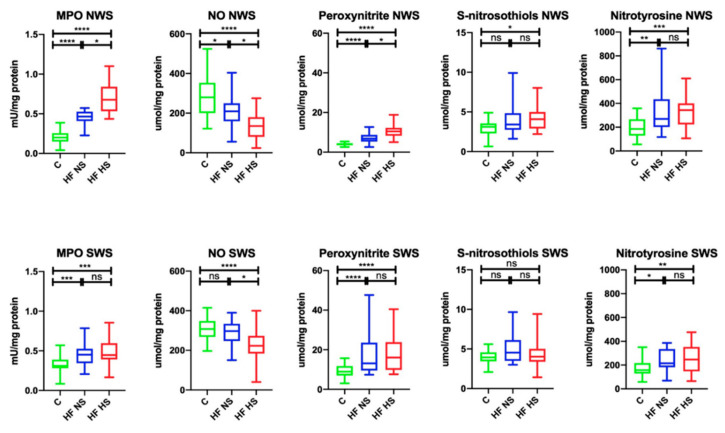
Salivary nitrosative stress in non-stimulated and stimulated saliva of HF patients and the control group. Abbreviations: HF NS—heart failure with normal salivation; HF HS—heart failure with hyposalivation; MPO—myeloperoxidase; NO—nitric oxide; NWS—non-stimulated whole saliva; SWS—stimulated whole saliva; * *p* < 0.05, ** *p* < 0.01, *** *p* < 0.001, and **** *p* < 0.0001.

In SWS, the activity of MPO (↑68.89%, *p* = 0.0005; ↑68.89, *p* = 0.0004, respectively) and the concentration of peroxynitrite (↑68.46%, *p* < 0.0001; ↑55.63%, *p* < 0.0001, respectively) and nitrotyrosine (↑73.15%, *p* = 0.0101; ↑63.97%, *p* = 0.0029, respectively) were significantly higher in the group of HF patients with NS and HS compared to the controls. NO concentration (↓72.4%, *p* < 0.0001) was considerably lower in HF patients with HS than in the control group. Within the study group, only NO concentration (↓75.08%, *p* = 0.0156) revealed a statistically significant difference expressed as its decreased level in HF patients with HS compared to HF subjects with NS (Figure 4).

### 3.6. Plasma Antioxidants

In the studied plasma samples of HF patients, UA concentration (↑51.02%, *p* < 0.0001; ↑60.24%, *p* = 0.0002, respectively) was significantly higher, while GSH concentration (↓72.09, *p* < 0.0001; ↓79.07%, *p* = 0.0017, respectively) was considerably lower in HF patients with NS, as well as HF subjects with HS, compared to the control group. Similar statistically significant different results were obtained for the levels of UA (↑52.08%, *p* < 0.0001; ↑61.73%, *p* = 0.0004, respectively) and GSH (↓68.65, *p* < 0.0001; ↓83.72%, *p* = 0.0161, respectively) in patients with New York Heart Association (NYHA) class II and III compared to the control group (Table 3).

### 3.7. Plasma Redox Status

In the plasma of HF patients, DPPH (↓73.06%, *p* = 0.0006; ↓70.06%, *p* = 0.0013, respectively) and FRAP (↓78.43%, *p* < 0.0001; ↓78.43%, *p* = 0.0002, respectively) were significantly lower in the group of HF patients with NS and HS compared to the controls, similarly to NYHA class II and III patients (DPPH: ↓77.03%, *p* = 0.0001; ↓76.46%, *p* = 0.0079, respectively, and FRAP: ↓78.43%, *p* < 0.0001; ↓80.39%, *p* = 0.0003, respectively) (Table 3).

### 3.8. Plasma Glycoxidation Products

The content of dityrosine (↑56.38%, *p* < 0.0001; ↑57.40%, *p* < 0.0001, respectively), kynurenine (↑65.38%, *p* < 0.0001; ↑64.56%, *p* < 0.0001), and glycophore (↑44.90%, *p* < 0.0001; ↑56.41%, *p* < 0.0001, respectively) was significantly higher in the group of HF patients with NS, as well as HS, compared to the control group, while the level of tryptophan (↓92.12%, *p* = 0.0114) was significantly lower in HF subjects with HS compared to the controls.

Similar differences were observed when comparing the content of dityrosine (↑59.88%, *p* < 0.0001; ↑60.42%, *p* < 0.0001, respectively), kynurenine (↑65.38%, *p* < 0.0001; ↑64.56%, *p* < 0.0001, respectively), and glycophore (↑45.83%, *p* < 0.0001; ↑56.41%, *p* < 0.0001, respectively) in NYHA class II and III groups compared to healthy controls, while the content of N-formylkynurenine (↑73.08%, *p* = 0.009) was significantly higher in NYHA class III patients compared to the control group, and tryptophan (↓90.40%, *p* = 0.0135) was considerably lower in this group compared to the controls (Table 3).

### 3.9. Plasma Nitrosative Stress

MPO activity (in both cases: ↑72.72%, *p* < 0.0001) and nitrotyrosine concentration (↑83.04%, *p* = 0.0039; ↑88.08%, *p* = 0.0044, respectively) were statistically significantly higher in the groups of HF patients with NS, as well as HS, compared to the controls, while the content of S-nitrosothiols (↓78.45%, *p* = 0.0008; ↓75.96%, *p* < 0.0001, respectively) was markedly lower. NO concentration (↑75.37%, *p* < 0.0001; ↑70.40%, *p* < 0.0001, respectively) was considerably higher in the HF group with NS compared to healthy controls and HF patients with HS.

Similar changes were noted when comparing MPO activity (↑72.72%, *p* < 0.0001 in both cases) and nitrotyrosine concentration (↑84.67%, *p* = 0.006; ↑87.66%, *p* = 0.0024, respectively) in NYHA class II and III groups compared to the control group, while the content of S-nitrosothiols (↓76.92%, *p* = 0.0002; ↓78.85%, *p* = 0.0005, respectively) was significantly lower. NO concentration (↑78.16%, *p* < 0.0001; ↑73.25%, *p* = 0.0014, respectively) was considerably higher in the NYHA class II group compared to the controls and NYHA class III patients (Table 3).

### 3.10. Salivary Antioxidants

Both in NWS and SWS, the total polyphenol content (NWS: ↓57.72%, *p* < 0.0001; ↓32.68%, *p* < 0.0001; SWS: ↓64.00%, *p* < 0.0001; ↓45.56%, *p* < 0.0001, respectively) and the concentrations of AA (NWS: ↓77.78%, *p* = 0.0027; ↓50%, *p* < 0.0001; SWS: ↓75%, *p* < 0.0001; ↓66.18%, *p* < 0.0001, respectively), GSH (NWS: ↓46.43%, *p* < 0.0001; ↓25.36%, *p* < 0.0001; SWS: ↓69.09%, *p* = 0.0012; ↓72.73%, *p* = 0.0006, respectively), and albumins (NWS: ↓74.19%, *p* = 0.0123; ↓48.39%, *p* < 0.0001; SWS: ↓44.11%, *p* = 0.0012; ↓47.06%, *p* = 0.0006, respectively) were significantly lower in NYHA class II and NYHA class III patients compared to the control group.

In NWS of the study group, TPC (↑56.62, *p* = 0.0492) and the levels of AA (↑64.29%, *p* = 0.0103) and albumins (↑65.22%, *p* = 0.0129) were considerably higher in NYHA class II patients compared to NYHA class III subjects.

UA concentration (↑68.38%, *p* = 0.0008) in NWS was significantly higher only in patients with NYHA class III compared to the control group, and, in SWS (↑75.46%, *p* = 0.0009; ↑78.02%, *p* = 0.0052, respectively), it was considerably higher in patients from both study groups (Table 4).

### 3.11. Salivary Redox Status

In NWS and SWS, DPPH (NWS: ↓77.03%, *p* = 0.0006; ↓36.56%, *p* < 0.0001; SWS: ↓55.18%, *p* < 0.0001; ↓26.89%, *p* < 0.0001, respectively) and FRAP (NWS: ↓68.18%, *p* = 0.0001; ↓62.12%, *p* < 0.0001; SWS: ↓84.06%, *p* < 0.0001; ↓73.91%, *p* = 0.0003, respectively) were significantly lower in the group of NYHA class II and class III patients compared to the control group (Table 4).

### 3.12. Salivary Glycoxidation Products

In the tested NWS and SWS samples, the levels of dityrosine (NWS: ↑76.71%, *p* < 0.0001; ↑71.34%, *p* < 0.0001; SWS: ↑84.84%, *p* = 0.0026; ↑83.05%, *p* = 0.0031, respectively), kynurenine (NWS: ↑76.92%, *p* < 0.0001; ↑78.95%, *p* = 0.0004; SWS: ↑82.14%, *p* = 0.0053; ↑74.19%, *p* = 0.0041, respectively) and glycophore (NWS: ↑81.3%, *p* < 0.0001; ↑65.79%, *p* < 0.0001; SWS: ↑73.38%, *p* = 0.0002; ↑56.04%, *p* < 0.0001, respectively) were significantly higher in NYHA class II and III compared to the control group, while tryptophan content (NWS: ↓77.55%, *p* = 0.036; SWS: ↓78.86%, *p* = 0.046) was considerably lower in NYHA class III patients compared to healthy controls. The content of N-formylkynurenine (↑61.88%, *p* < 0.0001; ↑49.5%, *p* < 0.0001, respectively) was significantly higher in NWS of NYHA class II and III patients compared to the control group, and, in SWS (85.71%, *p* = 0.0482), it was only higher in NYHA class II patient vs. the controls (Table 4).

### 3.13. Salivary Nitrosative Stress

In NWS and SWS, MPO activity (NWS: ↑42.55%, *p* < 0.0001; ↑28.57%, *p* < 0.0001; SWS: ↑67.39%, *p* < 0.0001; ↑68.89%, *p* = 0.002, respectively), as well as the concentration of peroxynitrite (NWS: ↑57.75%, *p* < 0.0001; ↑39.81%, *p* < 0.0001; SWS: ↑67.94%, *p* < 0.0001; ↑54.27%, *p* = 0.0001, respectively) and nitrotyrosine (NWS: ↑67. 77%, *p* = 0.0008; ↑53.02%, *p* = 0.0001; SWS: ↑67.22%, *p* = 0.0079; ↑64.13%, *p* = 0.0034, respectively), were significantly higher in the NYHA class II and III group compared to the control, while the concentration of S-nitrosothiols (↑83.78%, *p* = 0.033; 86.11%, *p* = 0.0487) was considerably higher only in NWS. NO concentration (↓48.52%, *p* = 0.0021; ↓70.35%, *p* < 0.0001, respectively) was notably lower in NWS of NYHA class II and III patients compared to the control group, while, in SWS (↓72.25%, *p* = 0.0002), only in patients with NYHA class III. Within the study group, statistically significant differences were expressed only as increased NO concentration (↑77.87%, *p* = 0.0424) in patients with NYHA class II compared to those with NYHA class III (Table 4).

### 3.14. Correlations

Correlations between salivary redox biomarkers and the activity of salivary glands are presented in Table 5.

In general, the content of redox biomarkers in the control group did not correlate with salivary gland activity. However, in NWS of HF patients with HS, we observed statistically significant correlations between flow rate (FR) and total protein (TP), as well as SA, and all the performed assays. Among salivary antioxidants, we obtained positive correlations between FR and TPC, AA, GSH, and albumins, between TP and TPC, AA, GSH, and albumins, and between SA and TPC, AA, GSH, and albumins. Negative correlations occurred between FR and UA, TP, and UA, as well as SA and UA. In the assays covering salivary redox status (DPPH, FRAP), we found a significant positive correlation between FR and TP, as well as SA, and DPPH and FR, and between TP, as well as SA, and FRAP. The assayed salivary glycoxidation products: dityrosine, kynurenine, N-formylkynurenine, and glycophore correlated negatively with FR, TP, and SA, while tryptophan correlated positively. Salivary nitrosative stress markers (MPO, peroxynitrite, S-nitrosothiols, nitrotyrosine) correlated negatively with FR, TP, and SA, and only NO correlated positively with them.

In NWS of HF patients with NS, only negative correlations are noteworthy: between TP and AA, albumins, FRAP, and N-formylkynurenine.

In stimulated saliva (SWS), we observed much fewer statistically significant correlations. Strong considerable correlations worth emphasizing in the group of HF patients with NS are: positive correlations between FR and TPC, TP and dityrosine, and between SA and AA, GSH, DPPH, dityrosine, kynurenine, N-formylkynurenine, MPO, peroxynitrite, S-nitrosothiols, and nitrotyrosine. In HF subjects with HS, only FR correlated negatively with AA and S-nitrosothiols.

### 3.15. ROC Analysis

The assessment of diagnostic usefulness of salivary antioxidants, redox status, glycoxidation products, and nitrosative stress biomarkers is presented in Table 6.

Particularly noteworthy are the evaluations of GSH concentration, N-formylkynurenine content, and MPO activity in NWS, allowing for high sensitivity and specificity in differentiating patients with NYHA class II and NYHA class III HF.

## 4. Discussion

Reduced saliva secretion is a common problem in people with chronic diseases [25,54]. Numerous studies have shown that oxidative/nitrosative stress plays a key role in salivary gland hypofunction in the course of systemic diseases [14,15,18,23]. However, still little is known about the mechanisms that lead to impairment of the salivary gland secretory function in HF patients. In this study, we were the first to compare the antioxidant barrier, protein glycoxidation and nitrosative/nitrative stress in HF patients with normal saliva (NS) secretion in comparison with HF subjects with HS. We demonstrated that salivary reserves of low-molecular-weight antioxidants (LMWA) are depleted in HF patients with salivary gland hypofunction, which may boost the glycoxidation and nitration/nitrosylation of salivary proteins. Interestingly, the concentration of most salivary redox biomarkers correlated negatively with the secretory activity of salivary glands.

The antioxidant defense of saliva includes both antioxidant enzymes (e.g., catalase, salivary peroxidase, superoxide dismutase) and non-enzymatic compounds (e.g., uric acid, UA; ascorbic acid, AA; reduced glutathione, GSH; albumin and polyphenols). However, it is LMWA that play an important role in maintaining oral health [55,56]. Indeed, reactions of LMWA with ROS are less specific than those of antioxidant enzymes, which makes LMWA more versatile ROS scavengers. They can react with superoxide radical anion and hydrogen peroxide (that skipped the effect of enzymes), thus reducing the chances of the formation of a very reactive hydroxyl radical. Furthermore, by participating in the second line of defense against ROS, LMWA direct oxidation reactions towards termination [55]. In our study, we observed decreased content of LMWA (↓TPC, ↓AA, ↓GSH, ↓albumin) in NWS of HF patients with HS compared to HF subjects with normal saliva (NS) secretion and to the controls (except albumin). Only the UA content in NWS was significantly higher in HF patients with HS compared to the other groups. However, this fact should not be surprising as hyperuricemia is commonly observed in HF patients [57,58,59], and the UA concentration in saliva generally reflects uric acid content in plasma [11,60]. Although UA represents 70–80% of the salivary antioxidant potential, this compound, when at high concentrations, has a strong prooxidant effect. Therefore, in our study, we additionally evaluated the total antioxidant activity of saliva by measuring DPPH and FRAP. These parameters provide information on the resultant capacity of free radical scavenging, considering the interactions between individual antioxidants [13,61]. Salivary DPPH and FRAP were significantly lower in SWS of HF patients compared to the controls, as well as considerably reduced in NWS of HF patients with HS compared to the other groups. This suggests the exhaustion of salivary antioxidant reserves in HF patients, which may result from increased ROS production. An important source of free radicals in the oral cavity is myeloperoxidase (MPO) that acts as a catalyst in the formation of hydrochloric acid (HOCl) in the reaction of Cl^−^ ion oxidation by hydrogen peroxide [62,63]. In the subsequent reaction, HOCl reacts with a superoxide radical anion to form an extremely reactive hydroxyl radical [63]. In our study, MPO activity was significantly higher in NWS of HF patients with HS compared to the other groups.

Decreased capacity of the antioxidant barrier may boost the oxidation/glycation of bio-molecules. The low probability of direct ROS reactions with lipids and DNA in the cell indicates that proteins are the primary target of oxygen and nitrogen free radicals. Indeed, in a typical eukaryotic cell, up to 70% of hydroxyl radicals react with proteins [64]. In our study, we observed increased concentration of protein glycoxidation products (↑dityrosine, ↑kynurenine, ↑glycophore) in NWS and SWS of HF patients with HS compared to the controls. Interestingly, the content of dityrosine, N-formylkynurenine, and glycophore in NWS was also significantly higher in HF patients with HS compared to HF subjects with normal saliva (NS) secretion. As for other systemic diseases, it can be assumed that the products of protein oxidation and glycation are aggregated and accumulated in the secretory cells of the salivary glands, which leads to progressive hypofunction of the glands. Protein glycoxidation products not only form a network of cross-links that disrupt the function of salivary gland cells, but they can also bind to a specific AGE receptor, thus increasing the production of ROS (by boosting NADPH oxidase activity) and inducing pro-inflammatory signaling pathways, e.g., NF-κB (nuclear factor kappa-light-chain-enhancer of activated B cells) or MAP kinases [65,66,67,68,69]. Under these conditions, the activity of proteasomes responsible for the removal of damaged proteins is impaired, which ultimately directs salivary gland cells to the apoptosis pathway [17]. In our study, the content of salivary glycoxidation products (except tryptophan) significantly negatively correlated with saliva secretion flow rate, total protein content and α-amylase activity, mainly in NWS of HF patients with HS. Generally, such a correlation was not found in patients with HF and normal saliva (NS) secretion, as well as in the control group. This may confirm our hypothesis on the role of protein oxidation/glycation in salivary gland dysfunction in HF patients. Moreover, LMWA content in NWS correlated positively (excluding UA) with the secretory function of salivary glands in HF patients with HS. Therefore, antioxidant supplementation should be considered to improve salivary gland activity in HF patients.

The process of saliva production consists of several stages [70]. In the first of them, the final section of the salivary gland secretory part (secretory acinus) produces primary saliva, which is similar in composition to the blood plasma. The isotonic primary saliva is then modified in the system of secretory ducts by selective reabsorption of Na^+^ and Cl^−^ ions, as well as secretion of K^+^ and HCO_3_^−^ [70,71]. These processes are initiated by the binding of various neurotransmitters to specific receptors on the surface of the secretory ducts and acini, which raises intracellular Ca^2+^ concentration [72]. An important role in this process is played by nitric oxide (NO), produced by neuronal nitric oxide synthase (nNOS), since it increases calcium ion concentration, thus triggering the activation of Ca^2+^-dependent potassium and chloride channels and starting the formation of primary saliva [70,73]. In our study, NO concentration was significantly lower in NWS and SWS of HF patients with HS compared to the control, and, in NWS, it was also lower compared to HF patients with normal saliva (NS) secretion. This indicates abnormal initiation of saliva secretion in patients with HF and HS. Decreased bioavailability of NO in these patients may be caused by boosted formation of peroxynitrite (ONOO^−^) in the reaction of nitric oxide with superoxide radical anion. Indeed, HF is accompanied by an overproduction of ONOO^−^, which is a strong oxidant, as well as a nitrating agent [31,32]. Peroxynitrite causes the nitration of aromatic amino acids (such as tryptophan and tyrosine), although the residues of sulfur-containing amino acid (such as cysteine and methionine) are the most susceptible to oxidation [74]. This fact can be confirmed by a negative correlation between peroxynitrite concentration in NWS and tryptophan and glutathione content in HF patients with HS. However, not only ONOO^−^ content was significantly higher in NWS of HF patients with HS compared to the controls, but also the concentration of the products of protein nitrosative modifications (↑S-nitrosothiols, ↑nitrotyrosine) was notably elevated in NWS of HF patients with HS, and it correlated negatively with the secretory function of salivary glands (saliva flow, total protein content, salivary amylase activity). It is believed that proteins damaged in this way accumulate mainly at the site of the formation of nitrating molecules [74]. The lack of correlation between nitrosative stress biomarkers in saliva and blood indicates a different nature of redox homeostasis disturbances at the local (salivary glands) and central (blood) level in HF patients. Furthermore, we found no correlation between saliva and blood in relation to other redox biomarkers (LMWA and glycoxidation products), which may confirm the local (oral cavity) response to free radical overproduction in HF patients.

The large salivary glands together produce about 90% of the total saliva volume. In our study, we found the weakening of the antioxidant barrier and increased glycoxidation/nitration of salivary proteins mainly in NWS of HF patients with HS. Since the submandibular salivary glands are primarily responsible for the secretion of non-stimulated saliva (they produce up to 2/3 of NWS total volume) [70], HF patients suffer from the hypofunction of this gland, in particular. However, in addition to oxidative/nitrosative stress, salivary gland dysfunction in HF patients may also result from damage to the salivary response and changes in the integrity of receptors in the gland tissues, as well as disorders in membrane transport and synthesis of proteins and their release into the saliva [75]. Therefore, this issue requires further research and clinical observation. Moreover, the influence of comorbidities on salivary gland function in HF patients cannot be excluded.

Many studies have shown that the oral health status of HF patients is very poor [76,77,78,79]. In this group, an increased incidence of dental caries and periodontal disease is observed. Interestingly, the inflammatory factor is vital in the initiation and progression of cardiovascular disorders: ischemic heart disease, arteriosclerosis, and acute coronary events, including myocardial infarction. In periodontitis, there is a local increase in the concentration of inflammatory mediators (IL-1, IL-6, and TNF-α), which not only have a destructive effect on the periodontium but can also initiate the formation of atherosclerotic plaque [77,78,80,81].

Unfortunately, we cannot eliminate the influence of pharmacotherapy on saliva secretion and composition. It is estimated that over 500 medicinal substances available on the pharmaceutical market may cause dry mouth symptoms. Additionally, the risk of such symptoms increases with the number of drugs taken [24,82]. In our study, HF patients received mainly beta blockers, diuretics, and statins. These medicines, by acting peripherally on alpha- and beta-adrenergic/cholinergic receptors and influencing electrolyte flow, can change the quantitative and qualitative composition of saliva [83,84,85]. Patients usually do not report any oral mucosa changes during the initial period of reduced salivary secretion. Advanced HS, on the other hand, results in dryness with the smooth, shiny, or wrinkled oral mucosa, atrophic lesions with smoothing or crushing of the papillae of the tongue, persistent and annoying burning of the mucous membrane of the tongue and lips (BMS, burning mouth syndrome), and rupture of the corners of the mouth with a tendency to inflammation, ulcers, and secondary fungal-bacterial infections [24,25,82]. In the case of polypharmacotherapy, the possibility of drug interactions affecting salivary gland function cannot be excluded [86].

Numerous studies have indicated that the overproduction of reactive forms of oxygen and nitrogen is responsible for structural and functional changes in the course of myocardium inefficiency [29,87,88,89,90,91]. The excess of free radicals leads to the oxidation of cardiolipin, the key phospholipid of the mitochondrial membrane necessary for energy production processes. Mitochondrial dysfunction hinders the already reduced energy metabolism in HF patients and intensifies previous metabolic changes [89,92,93,94]. In addition, under oxidative stress conditions, the activity of ROS-dependent signal kinases, such as PKC (protein kinase C), MAPK (mitogen-activated protein kinases), and Ras proteins, is increased, which contributes to cardiac hypertrophy [95]. Since oxidative stress plays an important role in HF progression [6,29], we additionally compared salivary redox biomarkers according to the severity of the disease. Generally, patients with NYHA class II and NYHA class III experience a decrease in the antioxidant barrier capacity and protein glycoxidation/nitration rate in NWS and SWS compared to the control. However, we did not observe any significant differences between the different stages of the disease progression. Only by means of ROC analysis were we able to demonstrate that GSH, N-formylkynurenine, and MPO evaluated in non-stimulated saliva can, with high sensitivity and specificity, differentiate patients with NYHA class II from those with NYHA class III.

Numerous advantages of saliva as a diagnostic material are more and more frequently emphasized. Saliva collection is easy, painless, and non-invasive, which is particularly important for screening tests and assessment of the disease progression, as well as monitoring of treatment results. Furthermore, unlike blood, saliva is a non-infectious material and can be collected without the involvement of medical personnel [61]. As salivary redox biomarkers are increasingly used in the diagnosis of various systemic diseases (obesity, hypertension, chronic kidney disease, psoriasis, dementia) [11,13,14,15,17,18,19,20,21,22], further studies are needed to assess the usefulness of salivary oxidative/nitrosative stress parameters in a larger population of HF patients.

## 5. Conclusions

1. Patients with chronic heart failure (HF) develop salivary gland dysfunction, with the submandibular salivary gland being the most inefficient.
2. Redox homeostasis disorders in HF patients are different at the local (salivary glands) and central (blood) level.
3. Oxidative/nitrosative stress may be one of the mechanisms responsible for the impairment of salivary gland secretory function in HF patients. Antioxidant supplementation should be considered to improve salivary gland activity in HF patients.
4. Salivary redox biomarkers are a potential diagnostic tool in HF patients; however, further studies should be conducted on the matter in a larger population of such patients.

## Figures and Tables

**Table 1:** Clinical characteristics of heart failure (HF) patients and the control group.

Patient Characteristics		Control *n* = 50	HF NS *n* = 27	HF HS *n* = 23	ANOVA *p*
Demographic data					
Sex	Male n (%)	29 (58)	14 (58.33)	15 (57.69)	NA
	Female n (%)	21 (42)	13 (61.9)	8 (38.1)	
Age		66 (42–87)	64 (49–85)	71 (42–87)	0.3337
Blood count					
WBC (×10^3^/µL)		7.44 (6.6–8.38)	7.23 (4.02–11.62)	7.65 (4.5–12.12)	0.2232
RBC (×10^6^/µL)		4.50 (3.51–5.62)	4.62 (3.38–12.9)	4.3 (3.34–5.49)	0.1114
HGB (g/dL)		13.52 (6–19.09)	13.75 (11.2–16.3)	13 (10.2–15.6)	0.2691
HCT (%)		38.72 (32.49–46.8)	39.9 (31.6–47.4)	38.9 (31.8–46.2)	0.1628
MCV (fL)		90.33 (78.63–97.32)	91.2 (76.2–105)	90.7 (78.2–98.5)	0.8838
MCH (pg)		33.49 (26.94–39.18)	30.95 (24.2–38.2) ^a^	30.5 (25.2–33.7) ^a^	<0.0001
MCHC (g/dL)		34.62 (27.58–40.5)	37.4 (24.09–49) ^a^	30.8 (25.2–43.37) ^b^	0.0003
RDW-SW (fL)		45.59 (42.95–47.98)	45.75 (37.1–58.6)	47.6 (42.2–55.6)	0.0904
PLT (×10^3^/µL)		250 (217.7–272.8)	175 (123–334) ^a^	189 (152–399) ^a^	<0.0001
PCT (%)		0.22 (0.17–0.25)	1.04 (0.25–2.94)	0.27 (0.16–0.31)	0.0382
MPV (fL)		7.91 (7.43–8.21)	14.69 (4.37–21.15) ^a^	11.8 (9.3–14.12) ^ab^	<0.0001
PDW (fL)		13.62 (10.37–16.38)	17.22 (11.41–21.57) ^a^	14.4 (10–17.5) ^b^	<0.0001
P-LCR (%)		30.13 (21.66–35)	36.82 (28.03–47.25) ^a^	36.1 (19.6–47.17) ^a^	<0.0001
Blood biochemistry					
CRP (mg/L)		2.92 (2.39–3.47)	1.8 (0.2–6.5)	3.49 (0.6–9.2) ^b^	0.0122
Na^+^ (mmol/L)		137.9 (129.4–149.7)	139 (133–143)	137 (125–141)	0.1754
K^+^ (mmol/L)		4.21 (3.94–4.6)	4.66 (3.46–6.07) ^a^	4.71 (3.71–5.83) ^a^	<0.0001
Creatinine (mg/dL)		0.91 (0.66–1.5)	0.93 (0.74–1.37)	1.08 (0.72–2.34) ^ab^	0.0001
GFR (ml/min)		85.69 (1.5–100.6)	84.01 (74.56–88.72)	73.99 (65.45–83.97) ^a^	0.0282
TSH (µIU/mL)		1.05 (0.69–1.5)	1.06 (0.03–2.56)	1.3 (0.38–4.18) ^ab^	0.0014
FT3 (pg/mL)		2.26 (1.3–3.0)	2.44 (1.38–3.14)	2.33 (1.65–3.15)	0.4556
FT4 (ng/mL)		9.78 (1.5–10.12)	5.67 (0.34–12.48) ^a^	4.22 (0.91–9.2) ^ab^	<0.0001
Vit. D_3_ (ng/mL)		24.04 (1.5–35.14)	18.05 (8.3–34.6) ^a^	12.1 (6.8–32.4) ^a^	<0.0001
AST (IU/L)		21.12 (1.5–27.91)	22.5 (12–37)	20 (15–37)	0.03
ALT (IU/L)		13.55 (1.5–16.42)	16 (7–44) ^a^	18 (10–41) ^a^	<0.0001
Glucose (mg/dL)		92.01 (75–101.3)	95.5 (85–104) ^a^	91.49 (78–102.9)	0.044
NT-proBNP (pg/mL)		ND	1782 (34–3644)	3339 (742–6610) ^b^	NA
Heart function					
NYHA II/NYHA III n		–	24/3	6/17	NA
EF		ND	26 (12–35)	20 (10–30) ^b^	NA
RR (mmHg)	SBP	125 (120–129.4)	124 (94–170)	125 (102–156)	0.3317
	DBP	71.06 (52.23–80)	75 (45–100) ^a^	75 (56–89)	0.0318
Comorbidities					
Type 2 diabetes n (%)		6 (14)	7 (29.17)	7 (26.92)	NA
Cardiac dysrhythmia (atrial flutter and fibrillation) n (%)		–	8 (33.33)	7 (26.92)	NA
Coronary artery disease n (%)		–	8 (33.33)	10 (38.46)	NA
Myocardial infarction n (%)		–	3 (12.5)	2 (7.69)	NA
Hypertension n (%)		20 (40)	19 (79.17)	17 (65.38)	NA
Medications					
Medications	ASA n (%)	6 (12)	10 (41.67)	8 (30.77)	NA
	Alpha receptor blocker n (%)	0 (0)	3 (12.5)	3 (11.54)	NA
	Beta receptor blocker n (%)	5 (10)	10 (37.04)	10 (43.47)	NA
	Ca^2+^ channel blocker n (%)	3 (6)	8 (33.33)	7 (26.92)	NA
	AT1-receptor blocker n (%)	8 (16)	8 (29.63)	9 (34.62)	NA
	Diuretics n (%)	8 (16)	14 (51.85)	12 (52.17)	NA
	ACE n (%)	6 (12)	13 (48.15)	12 (52.17)	NA
	Cardiac glycosides n (%)	0 (0)	3 (12.5)	3 (11.54)	NA
	Organic nitrate *n* (%)	0 (0)	1 (4.17)	1 (3.85)	NA
	Statins *n* (%)	9 (18)	13 (48.15)	10 (43.48)	NA

Abbreviations: ACE—angiotensin-converting enzyme; ALT—alanine transferase; ASA—acetylsalicylic acid; AST—aspartate aminotransferase; CRP—c-reactive protein; DBP—diastolic blood pressure; EF—ejection fraction; FT3—free fraction of triiodothyronine; FT4—free fraction of thyroxine; GFR—glomerular filtration rate; HCT—hematocrit; HF HS—heart failure with hyposalivation; HF NS—heart failure with normal salivation; HGB—hemoglobin concentration; K—potassium; MCH—mean corpuscular hemoglobin; MCHC—mean corpuscular hemoglobin concentration; MCV—mean corpuscular volume; MPV—mean platelet volume; Na—sodium; NT-proBNP—N-amino terminal fragment of the prohormone B-type natriuretic peptide; NWS—non-stimulated whole saliva; PCT—procalcitonin; PDW—platelet distribution width; P-LCR—platelet large cell ratio; PLT—platelets; RBC—red blood cells; RDW-SD—red cell distribution width, standard deviation; RR—blood pressure; SBP—systolic blood pressure; TSH—thyroid-stimulating hormone; WBC—white blood cells. ^a^ *p* < 0.05 vs. control, ^b^ *p* < 0.05 vs. HF NS.

**Table 2:** Salivary gland function and stomatological characteristics of HF patients and control subjects.

Patient Characteristics	Control *n* = 50	HF NS *n* = 27	HF HS *n* = 23
NWS FR (mL/min)	0.40 (0.31–0.53)	0.31 (0.21–0.46) ^a^	0.12 (0.001–0.19) ^ab^
SWS FR (mL/min)	1.3 (1.05–1.47)	0.8 (0.2–1.7) ^a^	0.6 (0.2–1.5) ^a^
NWS TP (μg/mL)	1390 (464.2–2107)	1230 (381.6–1821)	882 (486.5–1273) ^ab^
SWS TP (μg/mL)	1002 (125.4–1517)	1060 (347.1–1507)	863.2 (528.2–1174) ^ab^
NWS SA (µmol/mg protein)	0.18 (0.05–0.41)	0.12 (0.02–0.19) ^a^	0.05 (0.007–0.18) ^ab^
SWS SA (µmol/mg protein)	0.25 (0.09–0.81)	0.19 (0.1–0.33) ^a^	0.15 (0.08–0.26) ^a^
DMFT	28.62 (28.09–29.15)	28.96 (28.27–29.65)	29.09 (28.2–29.98)
GI	1.10 (0.4–1.17)	1.8 (1.53–2.07)	1.9 (1.66–2.14)
PBI	1.62 (1.56–1.68)	1.65 (1.51–1.79)	1.67 (1.53–1.81)

Abbreviations: DMFT—decayed, missing, filled teeth index; FR—flow rate; GI—gingival index; *n*—number of patients; HF HS—heart failure with hyposalivation; HF NS—heart failure with normal salivation; NWS—non-stimulated saliva; PBI—papilla bleeding index; SA—salivary amylase; SWS—stimulated saliva; TP—total protein. ^a^ *p* ˂ 0.05 vs. the control; ^b^ *p* ˂ 0.05 vs. HF NS.

**Table 3:** Plasma and erythrocyte redox biomarkers in HF patients and the control group.

	C *n* =50	HF NS *n* =27	HF HS *n* =23	ANOVA *p*-Value	C *n* =50	NYHA II *n* =30	NYHA III *n* = 20	ANOVA *p*-Value
Salivary antioxidants								
AA (μg/mg protein)	15.9 (3.0–27.5)	12.9 (2.6–27.6)	15.1 (0.58–33.7)	0.1741	15.9 (3.981–27.5)	13.4 (2.5–33.7)	12.4 (0.58–29.8)	0.2575
UA (μg/mg protein)	0.50 (0.18–0.98)	0.98 (0.24–1.3) ^a^	0.83 (0.38–1.1) ^a^	<0.0001	0.50 (0.18–0.98)	0.96 (0.24–1.3) ^a^	0.81 (0.38–1.1) ^a^	<0.0001
GSH (μg/mg protein)	4.3 (2.5–5.4)	3.1 (1.6–5.4) ^a^	3.4 (2.0–6.8) ^a^	<0.0001	4.3 (2.5–5.4)	3.0 (1.6–5.4) ^a^	3.6 (2.0–6.8) ^a^	<0.0001
Albumin (mg/mg protein)	2.4 (0.35–4.5)	3.3 (0.63–4.5)	2.5 (0.75–6.1)	0.3427	2.4 (0.35–4.5)	3.25 (0.63–4.55)	2.5 (1.3–6.1)	0.4745
Salivary redox status								
DPPH (nmol/mg protein)	156.3 (94.3–221.6)	114.2 (24.2–205.1) ^a^	109.5 (63.8–180.3) ^a^	<0.0001	156.3 (94.3–221.6)	107.3 (24.2–205.1) ^a^	119.5 (63.8–180.3) ^a^	<0.0001
FRAP (µmol/mg protein)	0.51 (0.30–0.69)	0.40 (0.17–0.59) ^a^	0.40 (0.28–0.56) ^a^	<0.0001	0.51 (0.30–0.69)	0.40 (0.17–0.59) ^a^	0.41 (0.28–0.52) ^a^	<0.0001
Salivary glycoxidation products								
Dityrosine (AFU/mg protein)	19.0 (6.9–27.0)	33.7 (12.7–54.2) ^a^	33.1 (20.7–48.2) ^a^	<0.0001	20.0 (6.9–27.0)	33.4 (12.7–54.2) ^a^	33.1 (20.7–48.2) ^a^	<0.0001
Kynurenine (AFU/mg protein)	5.1 (2.9–6.8)	7.8 (6.2–10.0) ^a^	7.9 (6.1–9.3) ^a^	<0.0001	5.1 (2.9–6.8)	7.8 (6.1–10.0) ^a^	7.9 (6.2–9.0) ^a^	<0.0001
N-formylkynurenine (AFU/mg protein)	1.9 (0.46–5.2)	2.5 (0.96–4.1)	2.5 (0.41–5.9)	0.0209	1.9 (0.5–5.2)	2.4 (0.5–4.1)	2.6 (0.4–5.9) ^a^	0.009
Tryptophan (AFU/mg protein)	69.8 (58.3–90.2)	68.3 (48.3–96.2)	64.3 (56.1–73.6) ^a^	0.0114	69.8 (58.3–90.2)	68.2 (48.3–96.2)	63.1 (56.1–73.6) ^a^	0.0105
Glycophore (AFU/mg protein)	2.2 (0.56–3.4)	4.9 (2.4–6.9) ^a^	3.9 (2.0–5.7) ^a^	<0.0001	2.2 (0.56–3.4)	4.8 (2.4–6.9) ^a^	3.9 (2.0–5.7) ^a^	<0.0001
Salivary nitrosative stress								
MPO (mU/mg protein)	0.80 (0.62–0.98)	1.1 (0.63–1.2) ^a^	1.1 (0.98–1.4) ^a^	<0.0001	0.80 (0.62–0.98)	1.1 (0.63–1.4) ^a^	1.1 (1.0–1.2) ^a^	<0.0001
NO (µmol/mg protein)	97.0 (58.7–151.9)	128.7 (75.0–191.3) ^a^	90.6 (44.1–150.4) ^b^	<0.0001	97.0 (58.7–151.9)	124.1 (50.6–191.3) ^a^	90.9 (44.1–150.4) ^b^	<0.0001
Peroxynitrite (µmol/mg protein)	175.1 (67.6–256.7)	193.5 (80.1–322.8)	191.9 (123.7–297.8)	0.3259	175.1 (67.6–256.7)	193.2 (80.1–322.8)	195.1 (123.7–297.8)	0.2504
S-nitrosothiols (µmol/mg protein)	10.4 (6.2–15.0)	8.2 (2.5–12.7) ^a^	7.9 (2.5–13.1) ^a^	<0.0001	10.4 (6.2–15.0)	8.0 (2.5–12.7) ^a^	8.2 (3.2–13.1) ^a^	<0.0001
Nitrotyrosine (µmol/mg protein)	181.1 (114.4–234.4)	218.1 (99.5–330.7) ^a^	205.6 (113.7–307.2) ^a^	0.0005	181.1 (114.4–234.4)	213.9 (99.5–330.7) ^a^	206.6 (154.6–307.2) ^a^	0.0004

Abbreviations: AA—ascorbic acid; DPPH—2,2-diphenyl-1-picrylhydrazyl radical; C—the control; FRAP—ferric-reducing antioxidant power; GSH—reduced glutathione; HF HS—heart failure with hyposalivation; HF NS—heart failure with normal salivation; MPO—myeloperoxidase; NO—nitric oxide; NWS—non-stimulated whole saliva; NYHA II—class II in the New York Heart Association (NYHA) classification of heart failure; NYHA III—class III in the New York Heart Association (NYHA) classification of heart failure; Px—salivary peroxidase; SOD—superoxide dismutase-1; SWS—stimulated whole saliva; TPC—total polyphenol content; UA—uric acid. ^a^ *p* ˂ 0.05 vs. the control; ^b^ *p* ˂ 0.05 vs. HF NS and NYHA II.

**Table 4:** Comparison of salivary redox biomarkers in NYHA class II, as well as NYHA class III, HF patients and the control group.

	NWS				SWS			
	C *n* = 50	NYHA II *n* = 27	NYHA III *n* = 23	ANOVA *p*-Value	C *n* = 50	NYHA II *n* = 30	NYHA III *n* = 20	ANOVA *p*-Value
Salivary antioxidants								
TPC (µg /mg protein)	66.7 (47.0–91.6)	38.5 (11.8–68.7) ^a^	21.8 (8.1–38.3) ^ab^	<0.0001	88.9 (62.2–103.9)	56.9 (23.0–97.0) ^a^	40.5 (11.3–67.9) ^a^	<0.0001
AA (μg/mg protein)	5.4 (3.9–9.0)	4.2 (2.5–9.7) ^a^	2.7 (2.1–8.3) ^ab^	<0.0001	6.8 (4.1–8.8)	5.1 (3.0–11.2) ^a^	4.5 (2.2–8.1) ^a^	<0.0001
UA (μg/mg protein)	65.7 (41.2–81.8)	74.9 (19.6–176.1)	94.7 (24.6–187.9) ^a^	0.0012	101.5 (45.3–192.1)	134.5 (66.6–382.9) ^a^	130.1 (35.1–310.0) ^a^	0.0002
GSH (μg/mg protein)	2.8 (1.7–3.6)	1.3 (0.46–2.9) ^a^	0.71 (0.42–1.5) ^a^	<0.0001	1.1 (0.54–1.9)	0.76 (0.22–1.7) ^a^	0.80 (0.43–1.2) ^a^	<0.0001
Albumin (mg/mg protein)	0.31 (0.12–0.53)	0.23 (0.04–0.67) ^a^	0.15 (0.03–0.24) ^ab^	< 0.0001	0.34 (0.16–0.47)	0.15 (0.01–0.76) ^a^	0.16 (0.04- 0.34) ^a^	<0.0001
Salivary redox status								
DPPH (nmol/mg protein)	209.5 (125.3–331.5)	148.8 (35.7–252.0) ^a^	76.6 (15.7–255.3) ^a^	<0.0001	307.2 (207.3–450.2)	169.5 (28.3–404.4) ^a^	82.6 (21.9–284.6) ^a^	<0.0001
FRAP (µmol/mg protein)	0.66 (0.37–0.85)	0.45 (0.27–0.88) ^a^	0.41 (0.27–0.68) ^a^	<0.0001	0.69 (0.50–0.94)	0.58 (0.26–0.96) ^a^	0.51 (0.23–1.1) ^a^	<0.0001
Salivary glycoxidation products								
Dityrosine (AFU/mg protein)	11.2 (5.5–14.9)	14.6 (8.9–26.3) ^a^	15.7 (11.8–27.3) ^a^	<0.0001	19.6 (14.8–25.0)	23.2 (12.7–46.5) ^a^	23.6 (12.2–47.3) ^a^	0.0003
Kynurenine (AFU/mg protein)	3.0 (1.2–4.3)	3.9 (2.5–8.1) ^a^	3.8 (2.4–5.4) ^a^	<0.0001	4.6 (3.5–6.5)	5.6 (1.9–10.8) ^a^	6.2 (3.7–11.5) ^a^	0.0005
N-formylkynurenine (AFU/mg protein)	0.99 (0.39–1.6)	1.6 (0.91–2.8) ^a^	2.0 (1.4–3.0) ^a^	<0.0001	1.8 (1.1–2.5)	2.1 (1.1–4.1) ^a^	1.8 (0.99–3.8)	0.0357
Tryptophan (AFU/mg protein)	44.1 (27.6–60.7)	37.7 (10.2– 84.1)	34.2 (10.2–61.5) ^a^	0.029	63.4 (46.8–82.2)	57.7 (35.9–94.3)	50.0 (13.9–96.4) ^a^	0.0064
Glycophore (AFU/mg protein)	10.0 (8.3–12.4)	12.3 (8.8–23.0) ^a^	15.2 (11.1–20.8) ^a^	<0.0001	10.2 (8.6–12.6)	13.9 (3.4–25.6) ^a^	18.2 (7.1–23.9) ^a^	<0.0001
Salivary nitrosative stress								
MPO (mU/mg protein)	0.20 (0.04–0.39)	0.47 (0.23–0.77) ^a^	0.70 (0.46–1.1) ^a^	<0.0001	0.31 (0.08–0.57)	0.46 (0.21–0.77) ^a^	0.45 (0.17–0.86) ^a^	<0.0001
NO (µmol/mg protein)	279.9 (121.7–524.3)	196.9 (55.7–403.6) ^a^	135.8 (23.8–275.4) ^a^	<0.0001	307.8 (196.6–414.8)	285.6 (139.7–389.7)	222.4 (40.1–399.8) ^ab^	0.0004
Peroxynitrite (µmol/mg protein)	4.1 (2.6–5.4)	7.1 (2.6–18.8) ^a^	10.3 (5.0–16.6) ^a^	<0.0001	8.9 (3.0–15.7)	13.1 (7.4–47.6) ^a^	16.4 (7.6–40.5) ^a^	<0.0001
S-nitrosothiols (µmol/mg protein)	3.1 (0.64–4.9)	3.7 (1.6–9.9) ^a^	3.6 (2.2–8.0) ^a^	0.0099	3.9 (2.1–5.6)	4.5 (3.0–9.7)	4.1 (1.4–9.4)	0.0582
Nitrotyrosine (µmol/mg protein)	184.6 (55.8–358.4)	272.4 (116.7–861.2) ^a^	348.2 (106.5–610.7) ^a^	<0.0001	157.9 (59.1–350.6)	234.9 (65.2–475.9) ^a^	246.2 (109.2–411.2) ^a^	0.0006

Abbreviations: AA—ascorbic acid; DPPH—2,2-diphenyl-1-picrylhydrazyl radical; C—the control; FRAP—ferric-reducing antioxidant power; GSH—reduced glutathione; HF HS—heart failure with hyposalivation; HF NS—heart failure with normal salivation; MPO—myeloperoxidase; NO—nitric oxide; NWS—non-stimulated whole saliva; NYHA II—class II in the New York Heart Association (NYHA) classification of the heart failure; NYHA III—class III in the New York Heart Association (NYHA) classification of the heart failure; Px—salivary peroxidase; SOD—superoxide dismutase-1; SWS—stimulated whole saliva; TPC—total polyphenol content; UA—uric acid. ^a^ *p* ˂ 0.05 vs. the control; ^b^ *p* ˂ 0.05 vs. NYHA II.

**Table 5:** Correlations between salivary redox biomarkers and secretory function of salivary glands.

	NWS												SWS										
	C				HF NS				HF HS				C				HF NS				HF HS		
	FR	TP	SA	FR		TP	SA	FR		TP	SA	FR		TP	SA	FR		TP	SA	FR		TP	SA
Salivary antioxidants																							
TPC	−0.072 0.001	0.37 0.008	−0.089 0.537	−0.018 0.928		0.001 0.998	0.395 0.041	0.748 <0.0001		0.779 < 0.0001	0.802 <0.0001	0.13 0.369		0.132 0.359	0.455 0.001	0.999 <0.0001		0.014 0.945	−0.013 0.949	0.069 0.755		0.143 0.514	−0.025 0.911
AA	0.051 0.726	0.21 0.143	−0.112 0.44	−0.054 0.788		−0.466 0.014	−0.154 0.444	0.933 <0.0001		0.959 <0.0001	0.979 <0.0001	0.457 0.001		−0.338 0.016	0.099 0.492	0.133 0.507		0.344 0.079	0.837 <0.0001	−0.559 0.006		0.22 0.312	−0.206 0.347
UA	0.135 0.352	−0.029 0.84	0.16 0.267	0.15 0.455		−0.175 0.382	0.143 0.478	−0.847 <0.0001		−0.841 <0.0001	−0.869 <0.0001	−0.259 0.07		0.062 0.668	−0.056 0.699	−0.151 0.453		0.188 0.348	0.208 0.297	−0.106 0.629		−0.09 0.683	−0.149 0.497
GSH	−0.109 0.453	−0.154 0.287	0.003 0.983	0.011 0.957		−0.268 0.177	0.228 0.253	0.892 <0.0001		0.89 <0.0001	0.902 <0.0001	0.223 0.119		−0.119 0.411	0.104 0.474	0.142 0.481		0.282 0.154	0.431 0.025	−0.08 0.715		0.168 0.444	−0.23 0.286
Albumin	0.047 0.743	−0.149 0.3	0.214 0.136	−0.054 0.788		−0.385 0.047	−0.022 0.913	0.867 <0.0001		0.883 <0.0001	0.892 <0.0001	−0.446 0.001		0.204 0.156	0.016 0.912	0.175 0.382		0.258 0.195	0.37 0.058	−0.117 0.594		0.152 0.488	−0.171 0.435
Salivary redox status																							
DPPH	−0.235 0.1	−0.085 0.556	−0.061 0.675	0.277 0.163		−0.118 0.556	0.171 0.395	0.902 <0.0001		0.934 <0.0001	0.959 <0.0001	0.047 0.745		−0.021 0.883	−0.037 0.801	0.04 0.844		0.203 0.309	0.444 0.02	−0.238 0.274		0.031 0.89	−0.022 0.922
FRAP	−0.152 0.291	0.035 0.808	0.077 0.594	−0.005 0.978		−0.41 0.034	0.245 0.219	0.823 <0.0001		0.855 <0.0001	0.845 <0.0001	−0.081 0.575		−0.098 0.497	−0.08 0.582	−0.128 0.524		0.244 0.22	0.365 0.061	−0.206 0.345		0.283 0.191	−0.288 0.183
Salivary glycoxidation products																							
Dityrosine	0.075 0.603	−0.038 0.791	0.1 0.488	−0.052 0.797		−0.262 0.187	−0.172 0.39	−0.763 <0.0001		−0.863 <0.0001	−0.861 <0.0001	0.005 0.973		0.013 0.929	0.099 0.495	−0.053 0.793		0.56 0.002	0.58 0.002	−0.33 0.124		−0.285 0.188	−0.18 0.412
Kynurenine	−0.099 0.492	0.099 0.494	−0.163 0.259	−0.013 0.949		−0.282 0.154	0.143 0.447	-0.826 <0.0001		−0.837 <0.0001	−0.818 <0.0001	0.055 0.705		−0.287 0.043	−0.011 0.939	−0.038 0.849		0.347 0.076	0.412 0.033	−0.241 0.267		0.222 0.308	−0.029 0.897
N-formylkynurenine	0.039 0.789	0.026 0.859	−0.099 0.494	−0.026 0.897		−0.454 0.017	0.142 0.481	−0.867 <0.0001		−0.919 <0.0001	−0.954 <0.0001	−0.373 0.008		0.218 0.128	−0.066 0.648	0.155 0.439		0.257 0.196	0.773 <0.0001	0.046 0.838		−0.217 0.331	−0.111 0.662
Tryptophan	−0.126 0.383	0.174 0.226	0.097 0.502	0.044 0.828		−0.269 0.174	0.109 0.587	0.812 <0.0001		0.816 <0.0001	0.826 <0.0001	0.08 0.58		−0.174 0.226	−0.038 0.795	−0.256 0.198		−0.125 0.536	−0.032 0.873	−0.351 0.1		0.189 0.388	−0.239 0.272
Glycophore	−0.178 0.215	0.032 0.827	−0.2 0.164	0.009 0.964		−0.152 0.449	−0.452 0.018	−0.839 <0.0001		−0898 <0.0001	−0.878 <0.0001	0.044 0.76		0.081 0.578	0.081 0.575	−0.168 0.403		0.177 0.377	−0.338 0.085	−0.091 0.68		−0.08 0.717	0.304 0.158
Salivary nitrosative stress																							
MPO	0.215 0.134	−0.278 0.051	0.286 0.044	−0.15 0.455		−0.288 0.145	−0.281 0.156	−0.825 <0.0001		−0.786 <0.0001	−0.842 <0.0001	−0.28 0.49		0.24 0.094	0.191 0.184	−0.076 0.707		0.205 0.305	0.412 0.033	−0.36 0.092		−0.13 0.553	−0.045 0.837
NO	−0.225 0.116	−0.055 0.703	0.018 0.902	0.14 0.487		0.231 0.247	−0.288 0.145	0.815 <0.0001		0.849 <0.0001	0.885 <0.0001	0.268 0.06		0.057 0.697	0.038 0.796	0.102 0.613		−0.073 0.716	0.061 0.762	−0.28 0.196		0.093 0.673	0.183 0.404
Peroxynitrite	−0.05 0.728	0.045 0.754	−0.053 0.713	−0.209 0.296		−0.364 0.062	0.023 0.91	−0.766 <0.0001		−0.733 <0.0001	−0.778 <0.0001	−0.042 0.772		0.141 0.33	−0.237 0.097	0.263 0.185		0.495 0.009	0.515 0.006	−0.065 0.767		0.062 0.778	0.022 0.922
S-nitrosothiols	0.268 0.06	−0.253 0.076	0.062 0.667	−0.007 0.973		−0.253 0.202	−0.021 0.916	−0.813 <0.0001		−0.817 <0.0001	−0.842 <0.0001	−0.005 0.973		−0.11 0.447	0.172 0.231	−0.13 0.517		0.168 0.401	0.454 0.017	−0.583 0.003		0.125 0.568	−0.187 0.394
Nitrotyrosine	−0.002 0.99	−0.136 0.347	−0.122 0.399	0.045 0.825		0.311 0.114	0.09 0.656	−0.784 <0.0001		−0.832 <0.0001	−0.862 <0.0001	0.133 0.358		−0.03 0.837	0.185 0.198	0.205 0.306		0.115 0.567	0.51 0.007	0.1 0.65		−0.035 0.876	−0.01 0.964

Abbreviations: AA—ascorbic acid; DPPH—2,2-diphenyl-1-picrylhydrazyl radical; C—the control; FRAP—ferric-reducing antioxidant power; GSH—reduced glutathione; HF HS—heart failure with hyposalivation; HF NS—heart failure with normal salivation; MPO—myeloperoxidase; NO—nitric oxide; NWS—non-stimulated whole saliva; Px—salivary peroxidase; SOD—superoxide dismutase-1; SWS—stimulated whole saliva; TPC—total polyphenol content; UA—uric acid. ^a^ *p* ˂ 0.05 vs. the control; ^b^ *p* ˂ 0.05 vs. HF NS.

**Table 6:** Receiver operating characteristic (ROC) analysis of oxidative stress biomarkers in the non-stimulated and stimulated saliva of HF patients.

	NWS								SWS							
	AUC	95% Cl	*p*-Value	Cut-off	Sensitivity %	95% Cl	Specificity %	95% Cl	AUC	95% Cl	*p*-Value	Cut-off	Sensitivity %	95% Cl	Specificity %	95% Cl
Salivary antioxidants																
TPC (μg/mg protein)	0.79	0.6627 to 0.9106	0.0007	<26.08	65	43.29 to 81.88	67	48.78 to 80.77	0.78	0.6487 to 0.9079	0.0009	<51.26	75	53.13 to 88.81	73	55.55 to 85.82
AA (μg/mg protein)	0.79	0.6567 to 0.9233	0.0006	<3.091	75	53.13 to 88.81	77	59.07 to 88.21	0.68	0.5245 to 0.8322	0.0341	<4.602	60	38.66 to 78.12	60	42.32 to 75.41
UA (μg/mg protein)	0.65	0.4933 to 0.8067	0.0747	<81.11	65	43.29 to 81.88	63	45.51 to 78.13	0.51	0.3494 to 0.6773	0.8741	<132.4	55	34.21 to 74.18	53	36.14 to 69.77
GSH (μg/mg protein)	0.84	0.7312 to 0.9521	<0.0001	<0.9130	75	53.13 to 88.81	77	59.07 to 88.21	0.54	0.3772 to 0.7094	0.6066	<0.7691	45	25.82 to 65.79%	47	30.23 to 63.86
Albumin (mg/mg protein)	0.72	0.5743 to 0.8657	0.0089	<0.1631	70	48.10 to 85.45	70	52.12 to 83.34	0.54	0.3746 to 0.6987	0.6631	<0.1590	50	29.93 to 70.07	47	30.23 to 63.86
Salivary redox status																
DPPH (nmol/mg protein)	0.76	0.6122 to 0.9111	0.0019	<117.3	70	48.10 to 85.45	70	52.12 to 83.34	0.71	0.5645 to 0.8588	0.0119	<119.9	65	43.29 to 81.88	63	45.51 to 78.13
FRAP (µmol/mg protein)	0.71	0.5639 to 0.8528	0.0133	<0.4216	65	43.29 to 81.88	63	45.51 to 78.13	0.64	0.4789 to 0.7977	0.1002	<0.5474	60	38.66 to 78.12	60	42.32 to 75.41
Salivary glycoxidation products																
Dityrosine (AFU/mg protein)	0.66	0.5034 to 0.8066	0.0655	>15.08	55	34.21 to 74.18	57	39.20 to 72.62	0.53	0.3580 to 0.6920	0.7664	>23.40	55	34.21 to 74.18	53	36.14 to 69.77
Kynurenine (AFU/mg protein)	0.58	0.4220 to 0.7414	0.3319	<3.807	55	34.21 to 74.18	53	36.14 to 69.77	0.53	0.3608 to 0.6926	0.7514	>5.989	55	34.21 to 74.18	53	36.14 to 69.77
N-formylkynurenine (AFU/mg protein)	0.88	0.7999 to 0.9521	<0.0001	>1.165	77	59.07% to 88.21	76	62.59 to 85.70	0.51	0.3327 to 0.6883	0.902	<1.916	58	36.28 to 76.86	57	39.20 to 72.62
Tryptophan (AFU/mg protein)	0.57	0.4059 to 0.7241	0.4399	<36.55	55	34.21% to 74.18%	57	39.20% to 72.62%	0.67	0.5050 to 0.8283	0.0477	<55.12	60	38.66 to 78.12	60	42.32 to 75.41
Glycophore (AFU/mg protein)	0.72	0.5726 to 0.8574	0.0106	>14.33	65	43.29 to 81.88	63	45.51 to 78.13	0.69	0.5296 to 0.8404	0.0279	>15.54	60	38.66 to 78.12	60	42.32 to 75.41
Salivary nitrosative stress																
MPO (mU/mg protein)	0.87	0.7717 to 0.9716	<0.0001	>0.5324	75	53.13 to 88.81	77	59.07 to 88.21	0.52	0.3494 to 0.6872	0.8276	<0.4495	55	34.21 to 74.18	53	36.14 to 69.77
NO (µmol/mg protein)	0.69	0.5373 to 0.8360	0.0266	<165.3	70	48.10 to 85.45	70	52.12 to 83.34	0.71	0.5483 to 0.8650	0.0141	<236.7	70	48.10 to 85.45	77	59.07 to 88.21
Peroxynitrite (µmol/mg protein)	0.75	0.6150 to 0.8917	0.0026	>8.721	70	48.10 to 85.45	70	52.12 to 83.34	0.52	0.3485 to 0.6881	0.8276	>14.21	60	38.66 to 78.12	60	42.32 to 75.41
S-nitrosothiols (µmol/mg protein)	0.51	0.3435 to 0.6699	0.9369	>3.657	50	29.93 to 70.07	50	33.15 to 66.85	0.63	0.4662 to 0.7871	0.1323	<4.277	55	34.21 to 74.18	57	39.20 to 72.62
Nitrotyrosine (µmol/mg protein)	0.56	0.3942 to 0.7225	0.4882	>308.1	60	38.66 to 78.12	60	42.32 to 75.41	0.55	0.3801 to 0.7099	0.5929	>246.2	50	29.93 to 70.07	50	33.15 to 66.85

Abbreviations: AA—ascorbic acid; DPPH—2,2-diphenyl-1-picrylhydrazyl radical; C—the control; FRAP—ferric-reducing antioxidant power; GSH—reduced glutathione; MPO—myeloperoxidase; NO—nitric oxide; NWS—non-stimulated whole saliva; Px—salivary peroxidase; SOD—superoxide dismutase-1; SWS—stimulated whole saliva; TPC—total polyphenol content; UA—uric acid.

## Data Availability

The article contains complete data used to support the findings of this study.

## References

[1] Tanai E., Frantz S. (2015). Pathophysiology of heart failure. Compr. Physiol..

[2] Metra M., Teerlink J.R. (2017). Heart failure. Lancet.

[3] Ponikowski P., Voors A. (2017). 2016 Esc guidelines for the diagnosis and treatment of acute and chronic heart failure: The Task Force for the diagnosis and treatment of acute and chronic heart failure of the European society of cardiology (ESC): Developed with the special contribution. Russ. J. Cardiol..

[4] D’Oria R., Schipani R., Leonardini A., Natalicchio A., Perrini S., Cignarelli A., Laviola L., Giorgino F. (2020). The Role of Oxidative Stress in Cardiac Disease: From Physiological Response to Injury Factor. Oxid. Med. Cell. Longev..

[5] Galougahi K.K., Antoniades C., Nicholls S.J., Channon K.M., Figtree G.A. (2015). Redox biomarkers in cardiovascularmedicine. Eur. Heart J..

[6] Van der Pol A., van Gilst W.H., Voors A.A., van der Meer P. (2019). Treating oxidative stress in heart failure: Past, present and future. Eur. J. Heart Fail..

[7] Ziaeian B., Fonarow G.C. (2016). Epidemiology and aetiology of heart failure. Nat. Rev. Cardiol..

[8] Orso F., Fabbri G., Maggioni A. (2017). Pietro. Epidemiology of heart failure. Handbook of Experimental Pharmacology.

[9] Saleh J., Figueiredo M.A.Z., Cherubini K., Salum F.G. (2015). Salivary hypofunction: An update on aetiology, diagnosis and therapeutics. Arch. Oral Biol..

[10] Yuan A., Woo S.B. (2015). Adverse drug events in the oral cavity. Oral Surg. Oral Med. Oral Pathol. Oral Radiol..

[11] Maciejczyk M., Taranta-Janusz K., Wasilewska A., Kossakowska A., Zalewska A. (2020). A Case-Control Study of Salivary Redox Homeostasis in Hypertensive Children. Can Salivary Uric Acid be a Marker of Hypertension?. J. Clin. Med..

[12] Soukup M., Biesiada I., Henderson A., Idowu B., Rodeback D., Ridpath L., Bridges E.G., Nazar A.M., Bridges K.G. (2012). Salivary uric acid as a noninvasive biomarker of metabolic syndrome. Diabetol. Metab. Syndr..

[13] Maciejczyk M., Szulimowska J., Taranta-Janusz K., Werbel K., Wasilewska A., Zalewska A. (2019). Salivary FRAP as A Marker of Chronic Kidney Disease Progression in Children. Antioxidants.

[14] Maciejczyk M., Szulimowska J., Taranta-Janusz K., Wasilewska A., Zalewska A. (2020). Salivary Gland Dysfunction, Protein Glycooxidation and Nitrosative Stress in Children with Chronic Kidney Disease. J. Clin. Med..

[15] Zalewska A., Kossakowska A., Taranta-Janusz K., Zięba S., Fejfer K., Salamonowicz M., Kostecka-Sochoń P., Wasilewska A., Maciejczyk M. (2020). Dysfunction of Salivary Glands, Disturbances in Salivary Antioxidants and Increased Oxidative Damage in Saliva of Overweight and Obese Adolescents. J. Clin. Med..

[16] Chielle E.O., Casarin J.N. (2017). Evaluation of salivary oxidative parameters in overweight and obese young adults. Arch. Endocrinol. Metab..

[17] Zalewska A., Maciejczyk M., Szulimowska J., Imierska M., Błachnio-Zabielska A. (2019). High-Fat Diet Affects Ceramide Content, Disturbs Mitochondrial Redox Balance, and Induces Apoptosis in the Submandibular Glands of Mice. Biomolecules.

[18] Zalewska A., Ziembicka D., Żendzian-Piotrowska M., Maciejczyk M. (2019). The Impact of High-Fat Diet on Mitochondrial Function, Free Radical Production, and Nitrosative Stress in the Salivary Glands of Wistar Rats. Oxid. Med. Cell. Longev..

[19] Skutnik-Radziszewska A., Maciejczyk M., Fejfer K., Krahel J., Flisiak I., Kołodziej U., Zalewska A. (2020). Salivary Antioxidants and Oxidative Stress in Psoriatic Patients: Can Salivary Total Oxidant Status and Oxidative Status Index Be a Plaque Psoriasis Biomarker?. Oxid. Med. Cell. Longev..

[20] Skutnik-Radziszewska A., Maciejczyk M., Flisiak I., Krahel J., Kołodziej U., Kotowska-Rodziewicz A., Klimiuk A., Zalewska A. (2020). Enhanced Inflammation and Nitrosative Stress in the Saliva and Plasma of Patients with Plaque Psoriasis. J. Clin. Med..

[21] Choromańska M., Klimiuk A., Kostecka-Sochoń P., Wilczyńska K., Kwiatkowski M., Okuniewska N., Waszkiewicz N., Zalewska A., Maciejczyk M. (2017). Antioxidant defence, oxidative stress and oxidative damage in saliva, plasma and erythrocytes of dementia patients. Can salivary AGE be a marker of dementia?. Int. J. Mol. Sci..

[22] Klimiuk A., Maciejczyk M., Choromańska M., Fejfer K., Waszkiewicz N., Zalewska A. (2019). Salivary Redox Biomarkers in Different Stages of Dementia Severity. J. Clin. Med..

[23] Gerreth P., Maciejczyk M., Zalewska A., Gerreth K., Hojan K. (2020). Comprehensive Evaluation of the Oral Health Status, Salivary Gland Function, and Oxidative Stress in the Saliva of Patients with Subacute Phase of Stroke: A Case-Control Study. J. Clin. Med..

[24] Nonzee V., Manopatanakul S., Khovidhunkit S.O.P. (2012). Xerostomia, hyposalivation and oral microbiota in patients using antihypertensive medications. J. Med. Assoc. Thail..

[25] Närhi T.O., Meurman J.H., Ainamo A. (1999). Xerostomia and hyposalivation: Causes, consequences and treatment in the elderly. Drugs Aging.

[26] Maria V., Beniamino P., Andrea M., Carmen L. (2017). Oxidative stress, plasma/salivary antioxidant status detection and health risk factors. Asian J. Med. Sci..

[27] Meleti M., Cassi D., Vescovi P., Setti G., Pertinhez T.A., Pezzi M.E. (2020). Salivary biomarkers for diagnosis of systemic diseases and malignant tumors. A systematic review. Med. Oral Patol. Oral Cir. Bucal.

[28] Zhang C.Z., Cheng X.Q., Li J.Y., Zhang P., Yi P., Xu X., Zhou X.D. (2016). Saliva in the diagnosis of diseases. Int. J. Oral Sci..

[29] Chen Q.M., Morrissy S., Alpert J.S. (2017). Oxidative Stress and Heart Failure. Comprehensive Toxicology.

[30] Bertero E., Maack C. (2018). Metabolic remodelling in heart failure. Nat. Rev. Cardiol..

[31] Pacher P., Schulz R., Liaudet L., Szabó C. (2005). Nitrosative stress and pharmacological modulation of heart failure. Trends Pharmacol. Sci..

[32] Pacher P., Beckman J.S., Liaudet L. (2007). Nitric oxide and peroxynitrite in health and disease. Physiol. Rev..

[33] Klimiuk A., Zalewska A., Sawicki R., Knapp M., Maciejczyk M. (2020). Salivary Oxidative Stress Increases With the Progression of Chronic Heart Failure. J. Clin. Med..

[34] Borys J., Maciejczyk M., Krȩtowski A.J., Antonowicz B., Ratajczak-Wrona W., Jablonska E., Zaleski P., Waszkiel D., Ladny J.R., Zukowski P. (2017). The redox balance in erythrocytes, plasma, and periosteum of patients with titanium fixation of the jaw. Front. Physiol..

[35] Maciejczyk M., Kossakowska A., Szulimowska J., Klimiuk A., Knaś M., Car H., Niklińska W., Ładny J.R., Chabowski A., Zalewska A. (2017). Lysosomal Exoglycosidase Profile and Secretory Function in the Salivary Glands of Rats with Streptozotocin-Induced Diabetes. J. Diabetes Res..

[36] Fejfer K., Buczko P., Niczyporuk M., Ładny J.R., Hady H.R., Knaś M., Waszkiel D., Klimiuk A., Zalewska A., Maciejczyk M. (2017). Oxidative Modification of Biomolecules in the Nonstimulated and Stimulated Saliva of Patients with Morbid Obesity Treated with Bariatric Surgery. Biomed Res. Int..

[37] Knaś M., Maciejczyk M., Sawicka K., Hady H.R., Niczyporuk M., Ładny J.R., Matczuk J., Waszkiel D., Żendzian-Piotrowska M., Zalewska A. (2016). Impact of morbid obesity and bariatric surgery on antioxidant/oxidant balance of the unstimulated and stimulated human saliva. J. Oral Pathol. Med..

[38] Maciejczyk M., Szulimowska J., Skutnik A., Taranta-Janusz K., Wasilewska A., Wiśniewska N., Zalewska A. (2018). Salivary Biomarkers of Oxidative Stress in Children with Chronic Kidney Disease. J. Clin. Med..

[39] WHO (2013). Oral Health Surveys: Basic Methods.

[40] Lobene R.R., Mankodi S.M., Ciancio S.G., Lamm R.A., Charles C.H., Ross N.M. (1989). Correlations among gingival indices: A methodology study. J. Periodontol..

[41] Löe H. (1967). The Gingival Index, the Plaque Index and the Retention Index Systems. J. Periodontol..

[42] Bernfeld P. (1955). Amylases, alpha and beta. Methods Enzymol..

[43] Jagota S.K., Dani H.M. (1982). A new colorimetric technique for the estimation of vitamin C using Folin phenol reagent. Anal. Biochem..

[44] Griffith O.W. (1980). Determination of glutathione and glutathione disulfide using glutathione reductase and 2-vinylpyridine. Anal. Biochem..

[45] Janaszewska A., Bartosz G. (2002). Assay of total antioxidant capacity: Comparison of four methods as applied to human blood plasma. Scand. J. Clin. Lab. Investig..

[46] Benzie I.F.F., Strain J.J. (1996). The Ferric Reducing Ability of Plasma (FRAP) as a Measure of “Antioxidant Power”: The FRAP Assay. Anal. Biochem..

[47] Diplock A.T., Symons M.C., Rice-Evans C.A. (1991). Techniques in Free Radical Research. Laboratory Techniques in Biochemistry and Molecular Biology.

[48] Kalousová M., Zima T., Tesař V., Dusilová-Sulková S., Škrha J. (2005). Advanced glycoxidation end products in chronic diseases-Clinical chemistry and genetic background. Mutat. Res.-Fundam. Mol. Mech. Mutagen..

[49] Kruidenier L., Kuiper I., van Duijn W., Mieremet-Ooms M.A.C., van Hogezand R.A., Lamers C.B.H.W., Verspaget H.W. (2003). Imbalanced secondary mucosal antioxidant response in inflammatory bowel disease. J. Pathol..

[50] Borys J., Maciejczyk M., Antonowicz B., Krętowski A., Sidun J., Domel E., Dąbrowski J.R., Ładny J.R., Morawska K., Zalewska A. (2019). Glutathione Metabolism, Mitochondria Activity, and Nitrosative Stress in Patients Treated for Mandible Fractures. J. Clin. Med..

[51] Grisham M.B., Johnson G.G., Lancaster J.R. (1996). Quantitation of nitrate and nitrite in extracellular fluids. Methods Enzymol..

[52] Beckman J.S., Ischiropoulos H., Zhu L., van der Woerd M., Smith C., Chen J., Harrison J., Martin J.C., Tsai M. (1992). Kinetics of superoxide dismutase- and iron-catalyzed nitration of phenolics by peroxynitrite. Arch. Biochem. Biophys..

[53] Wink D.A., Kim S., Coffin D., Cook J.C., Vodovotz Y., Chistodoulou D., Jourd’heuil D., Grisham M.B. (1999). Detection of S-nitrosothiols by fluorometric and colorimetric methods. Methods Enzymol..

[54] Islas-Granillo H., Borges-Yañez S.A., de Navarrete-Hernández J.J., Veras-Hernández M.A., Casanova-Rosado J.F., Minaya-Sánchez M., Casanova-Rosado A.J., Fernández-Barrera M.Á., Medina-Solís C.E. (2019). Indicators of oral health in older adults with and without the presence of multimorbidity: A cross-sectional study. Clin. Interv. Aging.

[55] Nagler R.M., Klein I., Zarzhevsky N., Drigues N., Reznick A.Z. (2002). Characterization of the differentiated antioxidant profile of human saliva. Free Radic. Biol. Med..

[56] Knaś M., Maciejczyk M., Waszkiel D., Zalewska A. (2013). Oxidative stress and salivary antioxidants. Dent. Med. Probl..

[57] Ndrepepa G. (2018). Uric acid and cardiovascular disease. Clin. Chim. Acta.

[58] Iliesiu A., Campeanu A., Marta D., Parvu I., Gheorghe G. (2015). Uric Acid, Oxidative Stress and Inflammation in Chronic Heart Failure with Reduced Ejection Fraction. Rev. Rom. Med. Lab..

[59] Bergamini C., Cicoira M., Rossi A., Vassanelli C. (2009). Oxidative stress and hyperuricaemia: Pathophysiology, clinical relevance, and therapeutic implications in chronic heart failure. Eur. J. Heart Fail..

[60] Sautin Y.Y., Johnson R.J. (2008). Uric Acid: The Oxidant-Antioxidant Paradox. Nucleosides Nucleotides Nucleic Acids.

[61] Toczewska J., Maciejczyk M., Konopka T., Zalewska A. (2020). Total Oxidant and Antioxidant Capacity of Gingival Crevicular Fluid and Saliva in Patients with Periodontitis: Review and Clinical Study. Antioxidants.

[62] Żukowski P., Maciejczyk M., Waszkiel D. (2018). Sources of free radicals and oxidative stress in the oral cavity. Arch. Oral Biol..

[63] Hansson M., Olsson I., Nauseef W.M. (2006). Biosynthesis, processing, and sorting of human myeloperoxidase. Arch. Biochem. Biophys..

[64] Cai Z., Yan L.-J. (2013). Protein Oxidative Modifications: Beneficial Roles in Disease and Health. J. Biochem. Pharmacol. Res..

[65] Singh R., Barden A., Mori T., Beilin L. (2001). Advanced glycation end-products: A review. Diabetologia.

[66] Ott C., Jacobs K., Haucke E., Navarrete Santos A., Grune T., Simm A. (2014). Role of advanced glycation end products in cellular signaling. Redox Biol..

[67] Żukowski P., Maciejczyk M., Matczuk J., Kurek K., Waszkiel D., Zendzian-Piotrowska M., Zalewska A. (2018). Effect of N-Acetylcysteine on Antioxidant Defense, Oxidative Modification, and Salivary Gland Function in a Rat Model of Insulin Resistance. Oxid. Med. Cell. Longev..

[68] De Coutinho T.A., Turner S.T., Peyser P.A., Bielak L.F., Sheedy P.F., Kullo I.J. (2007). Associations of Serum Uric Acid With Markers of Inflammation, Metabolic Syndrome, and Subclinical Coronary Atherosclerosis. Am. J. Hypertens..

[69] Beal M.F. (2002). Oxidatively modified proteins in aging and disease. Free Radic. Biol. Med..

[70] Proctor G.B. (2016). The physiology of salivary secretion. Periodontol. 2000.

[71] Carpenter G.H. (2013). The Secretion, Components, and Properties of Saliva. Annu. Rev. Food Sci. Technol..

[72] Proctor G.B., Carpenter G.H. (2007). Regulation of salivary gland function by autonomic nerves. Auton. Neurosci..

[73] Lomniczi A., Suburo A.M., Elverdin J.C., Mastronardi C.A., Diaz S., Rettori V., McCann S.M. (1998). Role of nitric oxide in salivary secretion. Neuroimmunomodulation.

[74] Förstermann U. (2010). Nitric oxide and oxidative stress in vascular disease. Pflugers Arch. Eur. J. Physiol..

[75] Odedra K., Ferro A. (2008). Neurohormones and heart failure: The importance of aldosterone. Int. J. Clin. Pract..

[76] Shetty D., Dua M., Kumar K., Dhanapal R., Astekar M., Shetty D.C. (2012). Oral hygiene status of individuals with cardiovascular diseases and associated risk factors. Clin. Pract..

[77] Najafipour H., Malek Mohammadi T., Rahim F., Haghdoost A.A., Shadkam M., Afshari M. (2013). Association of oral health and cardiovascular disease risk factors “results from a community based study on 5900 adult subjects”. ISRN Cardiol..

[78] Sanchez P., Everett B., Salamonson Y., Ajwani S., Bhole S., Bishop J., Lintern K., Nolan S., Rajaratnam R., Redfern J. (2017). Oral health and cardiovascular care: Perceptions of people with cardiovascular disease. PLoS ONE.

[79] Mathews M.J., Mathews E.H., Mathews G.E. (2016). Oral health and coronary heart disease. BMC Oral Health.

[80] Toczewska J., Konopka T., Zalewska A., Maciejczyk M. (2020). Nitrosative Stress Biomarkers in the Non-Stimulated and Stimulated Saliva, as well as Gingival Crevicular Fluid of Patients with Periodontitis: Review and Clinical Study. Antioxidants.

[81] Wilson K., Liu Z., Huang J., Roosaar A., Axéll T., Ye W. (2018). Poor oral health and risk of incident myocardial infarction: A prospective cohort study of Swedish adults, 1973–2012. Sci. Rep..

[82] Muhvić-Urek M., Kovačević-Pavičić D., Antonić R., Prpić J., Bonifačić I., Glažar I., Pezelj-Ribarić S. (2016). Medications as risk factors for hyposalivation in the elderly. Oral Diseases.

[83] Nederfors T., Nauntofte B., Twetman S. (2004). Effects of furosemide and bendroflumethiazide on saliva flow rate and composition. Arch. Oral Biol..

[84] Prasanthi B., Kannan N., Patil R. (2014). Effect of diuretics on salivary flow, composition and oral health status: A clinico-biochemical study. Ann. Med. Health Sci. Res..

[85] Maciejczyk M., Gerreth P., Zalewska A., Hojan K., Gerreth K. (2020). Salivary Gland Dysfunction in Stroke Patients Is Associated with Increased Protein Glycoxidation and Nitrosative Stress. Oxid. Med. Cell. Longev..

[86] de Matos L.F., Pereira S.M., Kaminagakura E., Marques L.S., Pereira C.V., van der Bilt A., Pereira L.J. (2010). Relationships of beta-blockers and anxiolytics intake and salivary secretion, masticatory performance and taste perception. Arch. Oral Biol..

[87] Dubois-deruy E., Peugnet V., Turkieh A., Pinet F. (2020). Oxidative stress in cardiovascular diseases. Antioxidants.

[88] Chang Y.-T., Chang W.-N., Tsai N.-W., Huang C.-C., Kung C.-T., Su Y.-J., Lin W.-C., Cheng B.-C., Su C.-M., Chiang Y.-F. (2014). The Roles of Biomarkers of Oxidative Stress and Antioxidant in Alzheimer’s Disease: A Systematic Review. Biomed. Res. Int..

[89] Tsutsui H., Kinugawa S., Matsushima S. (2008). Oxidative stress and mitochondrial DNA damage in heart failure. Circ. J..

[90] Tsutsui H., Kinugawa S., Matsushima S. (2011). Oxidative stress and heart failure. Am. J. Physiol. Hear. Circ. Physiol..

[91] Trachtenberg B.H., Hare J.M. (2009). Biomarkers of Oxidative Stress in Heart Failure. Heart Fail. Clin..

[92] Bayeva M., Gheorghiade M., Ardehali H. (2013). Mitochondria as a therapeutic target in heart failure. J. Am. Coll. Cardiol..

[93] Tsutsui H., Kinugawa S., Matsushima S. (2008). Mitochondrial oxidative stress and dysfunction in myocardial remodelling. Cardiovasc. Res..

[94] Martín-Fernández B., Gredilla R. (2016). Mitochondria and oxidative stress in heart aging. Age.

[95] Marquez J., Lee S.R., Kim N., Han J. (2006). Rescue of heart failure by mitochondrial recovery. Int. Neurourol. J..

